# Exploring the relationship between vascular remodelling and tumour growth using agent-based modelling

**DOI:** 10.1371/journal.pcbi.1012967

**Published:** 2026-05-15

**Authors:** Nicholas Fan, Joshua A. Bull, Helen M. Byrne

**Affiliations:** 1 Wolfson Centre for Mathematical Biology, Mathematical Institute, University of Oxford, Oxford, United Kingdom; 2 Ludwig Institute for Cancer Research, Nuffield Department of Medicine, University of Oxford, Oxford, United Kingdom; Johns Hopkins University, UNITED STATES OF AMERICA

## Abstract

We develop a multiscale agent-based model (ABM) to investigate the effect that mechanical interactions between proliferating tumour cells and the surrounding vasculature have on the oxygen supply to the tumour microenvironment (TME), the tumour’s growth dynamics, and its response to radiotherapy. Our model extends existing models of tumour spheroid growth by incorporating vessel deformation due to mechanical forces between vessel walls and neighbouring tumour cells. These forces generate an effective pressure which compresses vessels, driving occlusion and pruning. This, in turn, leads to a hypoxic oxygen landscape which stimulates angiogenesis. A key feature of our model is the treatment of mechanical cell interactions with the tumour microenvironment, which we represent with two forces. The first is Stokes’ drag which is widely used in ABMs to represent resistance to cell movement. The second is a friction force which accounts for resistance due to the continual breaking and reforming of cell-extracellular matrix (ECM) adhesions. The importance of this friction force is demonstrated by numerical simulation. When Stokes’ drag dominates, pressure gradients dissipate across the tissue and vessel compression is negligible. By contrast, as the strength of the friction force increases, larger pressure gradients form, leading to significant vessel compression. We perform extensive numerical simulations to investigate how model parameters that control vascular remodelling and friction influence tumour vascularisation, which we spatially quantify using the cross-pair correlation function. This, in turn, alters the oxygen landscape and drives changes in tumour morphology. Finally, we highlight the importance of accounting for both mechanisms when simulating tumour responses to treatment with radiotherapy. We observe that vascular remodelling critically alters the tumour’s susceptibility to treatment and post-radiotherapy regrowth. Tumour regrowth is especially impacted by vessel remodelling, with certain vascular landscapes able to rebound quickly post-radiotherapy, resulting in fast tumour regrowth.

## Author summary

We have created an agent-based model (ABM) that accounts for mechanical interactions between tumour cells and associated vasculature. The model simulates vessel occlusion and pruning due to compression by neighbouring tumour cells; and growth of new vessels in response to hypoxia. Incorporating pressure-mediated vessel occlusion into our ABM required development of a friction force representing cell-extracellular matrix (ECM) adhesion. Through numerical simulations, we show that when friction is neglected, pressure gradients dissipate throughout the tissue and vessel compression is negligible. By contrast, as the strength of the friction force increases, larger pressure gradients form, leading to significant vessel compression. We perform extensive model simulations, to investigate how changes in the oxygen landscape caused by vascular remodelling affect the tumour’s growth dynamics and composition. We show further how vessel remodelling influences a tumour’s response to radiotherapy. We find tumour regrowth to be especially sensitive to vessel remodelling, with certain vascular landscapes growing rapidly post-radiotherapy, and accelerating tumour regrowth.

## 1. Introduction

Tumours contain a diverse collection of cell populations and cytokines, which, with the extracellular matrix, make up the tumour microenvironment (TME). TME composition varies between tumours, and its evolution is shaped by the constituent tumour cells, infiltrating immune cells, stromal cells, blood vessels and extracellular matrix [1–3]. Interactions between these species produce distinct TME landscapes which can have strong pro-tumour effects [4]. In this paper we develop a multiscale model to investigate the effect that mechanical interactions between proliferating tumour cells and the surrounding vasculature have on oxygen supply to the TME, the tumour’s growth dynamics, and its response to radiotherapy [5].

Tumour growth impacts blood vessels in two important ways. First, mechanical stress generated by rapid tumour growth increases the solid pressure that the tumour cells exert on blood vessels, causing them to become compressed and occluded, and reducing their blood flow [6,7]. Further, if the flow rate in a vessel remains sufficiently low for a sufficiently long period then the vessel may be pruned from the vascular network [8]. These factors limit the ability of the vasculature to supply oxygen to the surrounding tissue, leading to reduced oxygen levels which slow tumour growth [9]. This leads to the second effect, where low oxygen levels (hypoxia) stimulate tumour cells to produce diffusible factors such as vascular endothelial growth factor (VEGF) which promote the growth of new blood vessels through angiogenesis [10], one of Hanahan and Weinberg’s hallmarks of cancer [11–13]. The newly formed vessels are typically abnormal, and the associated blood flow is often unstable and highly irregular, which limits their ability to meet the tumour’s oxygen requirements [14,15].

In this paper, we develop a spatially-resolved model to investigate the effect that interactions between tumour cells and blood vessels have on their co-evolution. Spatially resolved mathematical models of tumour growth fall into two broad categories: continuum models, which describe the time evolution of the tumour density, and agent-based models (ABMs), which distinguish individual cells and whose evolution is governed by pre-defined rules. ABMs can be divided into on-lattice approaches, including cellular automata [16–18] and cellular Potts models [19–21]; and off-lattice approaches such as node-based models [22] and vertex models [23–25]. Hybrid models combine multiple approaches, for example treating certain species or regions as discrete agents (often cells) and others as continuous variables (often diffusible species). For reviews of these different modelling approaches, see [26–31].

We propose a hybrid model, which uses an off-lattice, node-based framework to resolve individual tumour cells and blood vessels and uses a reaction-diffusion equation to determine how the spatial distribution of oxygen evolves over time. This hybrid approach has been used in other models of tumour growth because of its flexibility (see, e.g., [22,32–40]). Our model builds upon these approaches by accounting for vessel compression caused by high solid pressure generated by tumour growth. This pressure causes deformation of the vessel wall, and drives vessel occlusion and pruning [6,7]. Existing ABMs that account for vessel occlusion typically focus on the wall shear stress experienced by blood vessels during blood flow and assume that the wall shear stress must exceed a threshold value if a given vessel is to remain viable and support blood flow: a vessel becomes occluded if the wall shear stress it experiences remains below the threshold value for a sufficiently long period [41–48]. This approach does not account for vessel remodelling due to mechanical pressure generated by tumour growth, an effect shown to be important in experiments [49] and continuum models [50–54].

A key feature of our ABM is the treatment of mechanical interactions between cancer cells and components of the surrounding TME, such as the extracellular matrix (ECM) and extracellular fluid. We account for these interactions with two distinct forces. The first is Stokes’ drag, widely used in ABMs to represent resistance to cell movement due to extracellular fluid. The second, a novel friction force, accounts for resistance to motion due to the continual breaking and formation of cell-ECM bonds of adhesion. While other models account for resistance to motion due to cell-ECM bonds of adhesions [55–57] by explicitly modelling the ECM and its deformation during cell movement, our approach instead models these effects as a combination of friction and drag. Through numerical simulation we demonstrate that friction is necessary for the generation of solid pressure gradients that are sufficiently large to drive vessel occlusion: when Stokes’ drag dominates, solid pressure gradients dissipate across the tissue and vessel compression is negligible. By contrast, as the strength of the friction force increases, larger solid pressure gradients form and drive vessel occlusion. While our model provides a mechanism to study the impact of solid pressure on vessel occlusion, we note that the effects of fluid pressure within the extracellular fluid are neglected explicitly.

We incorporate vessels into the ABM by building on earlier work in which blood vessels were represented as fixed point sources of oxygen, of negligible volume [32,33,38,58]. Here we view vessels as space-occupying agents which interact with tumour cells. While vessel locations are fixed, deformation of vessel walls is caused by forces exerted on them by tumour cells; put simply, the pressure difference across a vessel wall determines whether it is compressed (external pressure exceeds internal pressure) or expands (external pressure less than internal pressure). We assume further that changes in vessel cross-sectional area modulate the supply of oxygen to the tissue and the rate at which vessels are pruned from the system, a simplifying approximation which reflects oxygen tension varying between vessels of different sizes. We incorporate additional rules to account for the growth of new vessels in response to hypoxia.

We demonstrate the impact of including vascular remodelling and the friction force in the ABM by using it to simulate treatment with radiotherapy, a front-line treatment for cancer [59,60]. We show that the additional modelling assumptions in our ABM can have a significant effect on the tumour’s response to radiotherapy. Due to its prevalence in the clinic, there are many mathematical models of tumour responses to radiotherapy (for example, [61–66]). We follow [58,62,67], by simulating radiotherapy with the linear quadratic model. We show that even this simple implementation of radiotherapy, when integrated within our ABM, produces a complex treatment and recovery landscape.

Quantifying the intricate spatial structures generated by ABMs requires the application of spatial analysis methods. Here we use spatial, and shape metrics to describe and quantify a tumour’s vascularisation and morphology. Spatial statistics are commonly used in fields such as astrophysics and ecology and are ideally suited to describe, quantify and compare the location of individual cells in tissue images and ABMs [68–72]. These methods enable us to understand how a tumour and its vasculature evolve over time and as parameters relating to friction and vascular efficacy vary.

The remainder of this paper is structured as follows: in the next section, we summarise our model, focusing on how it extends previous work by Bull & Byrne [32,33]. We then describe our simulation protocol and parameter sweeps, before introducing the metrics we use to quantify simulation outputs. In the Results section, we demonstrate the importance of friction in enabling pressure to accumulate within a growing tumour and show that such behaviour cannot be generated when cell-ECM interactions are represented by drag forces alone. We investigate further the effect of pressure accumulation on the vasculature, showing how the strength of the friction force and vessel robustness to mechanical stress modulate the degree of vascularisation of a tumour. Vascular remodelling due to tumour growth in turn alters the oxygen landscape, and we show that if the tumour’s demand for oxygen exceeds the supply then this may drive morphological changes that result in multilobular tumours. Finally, we show that mechanically-mediated vascular remodelling affects tumour sensitivity to radiotherapy and the tumour’s subsequent recovery dynamics.

## 2. Methods

In this section we outline our agent-based model (ABM), which builds on an earlier model developed by us [33]. Here we focus on the new elements of the model (full model details are included in S1 Text). We then describe our simulation and parameter sweep protocol, and introduce the methods used to characterise and quantify the qualitative behaviours that the ABM generates.

### 2.1. Agent-based model

**Fig 1:**
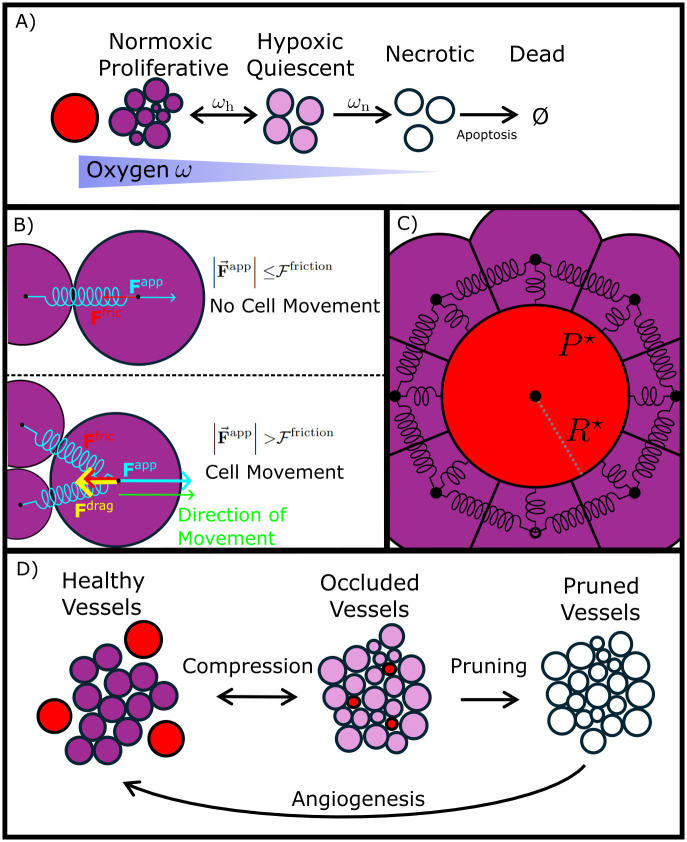
Schematic summarising the key mechanisms in the agent-based model. (A) Tumour cells (purple) consume oxygen which is supplied by blood vessels (red). Tumour cell behaviour is determined by the local oxygen concentration: ‘proliferative’ in well-oxygenated areas ($\omega_{h}<\omega$), ‘quiescent’ in intermediate oxygen levels ($\omega_{n}<\omega \leq \omega_{h}$) and ‘necrotic’ when oxygen is so low that tumour cells die due to lack of oxygen ($0\leq \omega \leq \omega_{n}$). (B) Cell movement is determined by balancing the forces acting on a cell. These include spring forces due to physical contact with neighbouring cells which drive movement, and a combination of friction and drag forces which resist movement. (C) Blood vessels experience forces, and in turn apply them to neighbouring tumour cells. The total pressure applied to a vessel is compared to its internal vessel pressure $P^{⋆}$. This pressure difference determines the dynamics of the vessel’s radius $R^{⋆}(t)$, allowing a vessel to be mechanically compressed or to expand. (D) Vessel phenotype is determined by its radius, $R^{⋆}(t)$ which depends on the local cell pressure it experiences: in low pressure the vessel is ‘healthy’. Under compression, it becomes ‘occluded’, and its oxygen supply decreases. If a vessel remains occluded for longer than $\tau_{\text{prune}}$, then it is pruned, and no longer acts as an oxygen source. The lack of oxygen stimulates angiogenesis, whereby a new vessel may form at the same location to restore the oxygen supply.

Our ABM is developed within CHASTE (Cancer, Heart and Soft Tissue Environment), an open source C++ framework for simulating complex, multiphysics, and multiscale mathematical models of biomedical systems, including cancer [73–75]. We adopt a hybrid approach to develop our 2D ABM, using an off-lattice, cell-centre based model to resolve individual tumour cells located within a dynamic, vascular tissue. Following [33], each cell possesses a subcellular cell-cycle model, which determines whether and when a cell proliferates. Proliferation depends on oxygen (ω), which is viewed as a diffusible species whose spatial distribution is modelled by a reaction-diffusion equation (Section S1.1.1 in S1 Text), with vessels as point sources and tumour cells as point sinks. As shown in Fig 1A, under normoxic conditions ($\omega \geq \omega_{h}$) tumour cells consume oxygen and proliferate. Under low oxygen conditions ($\omega_{n}<\omega \leq \omega_{h}$) they stop proliferating while continuing to consume oxygen (Section S1.1.2 in S1 Text). If the local oxygen concentration becomes too low ($\omega \leq \omega_{n}$), then the tumour cells become necrotic and die (via a process described in Section S1.1.3 in S1 Text). Tumour cells also stop proliferating if they are mechanically compressed, i.e., if their area is less than a proportion η of their target area. Cells are physically represented by their centroid and mechanical interactions with nearby cells are modelled via a system of linear springs that connect the cell centroids. Each spring has a target length and resistance which represent each cell’s uncompressed diameter and compressibility respectively. Cells exert forces on their neighbours in order to maintain their target spring lengths (Section S1.3.1 in S1 Text). Fig 1B shows how the balance of forces applied by cells and vessels acting on a cell is resolved and drives cell movement (Section S1.3.2 in S1 Text). For a detailed description, see S1 Text.

We now describe the new features of our ABM: vessel remodelling, including occlusion, pruning and angiogenesis; and a friction force which enables pressure to accumulate within the tumour.

#### 2.1.1. Vessel remodelling.

In [33], blood vessels are modelled as fixed point sources of oxygen which do not occupy space. Here, we instead represent them as dynamic agents which occupy space. As before, for simplicity, vessels are assumed to be perpendicular to the plane and, therefore, we represent them as circular agents whose radii evolve in response to environmental cues.

Below, we describe how vessels supply oxygen to the tissue at varying rates depending on their radius. Then we describe the different phenotypes a blood vessel may adopt and how transitions between phenotypes account for vessel pruning and angiogenesis. Later we explain the force balance which determines the evolution of a vessel’s radius in response to applied forces, see Section 2.1.3.

#### 2.1.2. Dynamic oxygen supply.

A vessel’s radius $R^{⋆}$ determines its rate of oxygen supply to the surrounding tissue. The oxygen distribution ω is modelled via a reaction diffusion equation of the form


$$\begin{array}{c} \frac{\partial \omega }{\partial t}=\underset{\text{Diffusion}}{\underbrace{D_{\omega }\nabla^{2}\omega }}+\underset{\text{Diffusion from blood Vessels}}{\underbrace{\alpha \left(1-\omega \right)\sum_{i\in \text{blood vessels}}\alpha_{i}\delta \left(\vec{𝐫}-\vec{r_{i}}\right)}}-\underset{\text{Consumption by cells}}{\underbrace{\kappa \omega \sum_{i\in \{\text{cells}\}}\delta \left(\vec{𝐫}-\vec{r_{i}}\right)}}-\lambda \omega , \end{array}$$ (1)

where the positive parameters $D_{\omega }$ and κ represent the oxygen diffusion coefficient and the rate at which cancer cells consume oxygen respectively. The decay rate λ accounts for oxygen consumption by cells not explicitly included in the model. In Equation (1), we assume that the oxygen concentration in the blood vessels is constant, normalised to unity, and that the rate at which the *i*-th blood vessel supplies oxygen to the environment is proportional to the difference between the oxygen concentrations in the blood vessel and the tissue, and denote by α the maximum rate of oxygen supply from a blood vessel. We introduce a scaling factor $\alpha_{i}$ to account for the dependence of the supply rate on the vessel radius *R*_*i*_. For simplicity, we assume that $\alpha_{i}\in [0,1]$ is a linearly increasing function of *R*_*i*_ of the form


$$\begin{array}{c} \alpha_{i}=\frac{R_{i}^{⋆}-{\hat{R}}_{\text{min}}}{{\hat{R}}_{\text{max}}-{\hat{R}}_{\text{min}}}, \end{array}$$ (2)

where the parameters ${\hat{R}}_{\text{min}}$ and ${\hat{R}}_{\text{max}}$ denote the minimum and maximum radii of a blood vessel. We close Equation (1) by imposing no-flux boundary conditions on the domain boundaries:


∂ω∂n=0 on the domain boundaries, where n is the normal to the boundary.

We assume that initially $\omega \left(\vec{𝐫},t=0\right)=1,\forall \;\vec{𝐫}\in {\mathbb{R}}^{2}$

#### 2.1.3. Occlusion, Pruning and Angiogenesis.

We distinguish three vessel phenotypes: **Healthy**, **Occluded** and **Pruned**. The **Healthy** and **Occluded** phenotypes are determined based on the vessel radius:


$$\begin{array}{c} \text{Vessel Phenotype}=\left\{ \begin{array}{cc} \text{Healthy} & \text{ if }R^{⋆}\geq {\hat{R}}_{\text{occ}}\\ \text{Occluded} & \text{ if }R^{⋆}<{\hat{R}}_{\text{occ}} \end{array}\right.. \end{array}$$ (3)

where ${\hat{R}}_{\text{occ}}$ is the threshold vessel radius at which a vessel becomes occluded.

**Healthy** and **Occluded** phenotypes behave similarly, both allow a vessel to supply oxygen to the tissue dependent on the vessel’s radius as described above. Vessels can also quickly transition between **Healthy** and **Occluded** phenotypes as their radius dynamically evolves due to applied forces (see Section 2.1.3). We distinguish the **Occluded** phenotype as vessels which are sufficiently compressed such that they are candidates to be pruned. This is modelled as a vessel’s phenotype switching to **Pruned** if they remain **Occluded** for longer than $\tau_{\text{prune}}$.

**Pruned** vessels are irreparably damaged, considered dead and have been removed from the simulation. **Pruned** vessels may only return to the **Healthy** phenotype following successful angiogenesis. We model angiogenesis by returning **Pruned** vessels to the **Healthy** phenotype under two conditions: First, there must be at least one **Healthy** vessel within distance *D*_angio_ of the **Pruned** vessel from which the new vessel can emerge. Secondly, the candidate location must be sufficiently hypoxic for an extended period of time. This is defined as $\omega <\omega_{\text{angio}}$ for longer than $\tau_{\text{angio}}$ and represents the time taken for tumour cells, stimulated by hypoxia, to produce angiogenic factors such as VEGF which stimulate vessel growth. If these two conditions are met then the vessel has probability of *P*_angio_ to regrow each hour. This is summarised by the pseudocode in Algorithm A in S1 Text. The overall system of vessel phenotypes and the transitions between them are summarised in Fig 1D.

We note that in practice, when a vessel is pruned, the surrounding vessels are likely to experience increased blood flow; for simplicity, this effect is neglected in the current model.

#### 2.1.4. Tumour cell force balance.

In existing cell-centre models the equations of motion are derived by balancing the applied forces ${\vec{𝐅}}^{\text{app}}$ acting on a cell with a Stokes’ drag force ${\vec{𝐅}}^{\text{drag}}$, which models the net effect of TME interactions that resist cell movement. Details on the applied contact force ${\vec{𝐅}}^{\text{app}}$ are given in Section S1.3.1 in S1 Text. In practice, a component of a cell’s interaction with the ECM is the formation of adhesive bonds. These attachments anchor a cell in place and prevent it from moving when small forces are applied; the cell only moves when the applied forces ${\vec{𝐅}}^{\text{app}}$ are sufficiently large to break the attachments. Stokes’ drag does not account for this effect, which we model with a friction force ${\vec{𝐅}}^{\text{fric}}$.

If $|{\vec{𝐅}}^{\text{app}}|<{ℱ}^{\text{friction}}$ then the applied force is exactly balanced by friction (${\vec{𝐅}}^{\text{fric}}=-{\vec{𝐅}}^{\text{app}}$) and the cell does not move. In this case, the friction force corresponds to a static friction force and the parameter ${ℱ}^{\text{friction}}$ represents the magnitude of the limiting friction force.

If $|{\vec{𝐅}}^{\text{app}}|\geq {ℱ}^{\text{friction}}$ then the limiting friction force is unable to balance the applied force and the cell moves. In this case, we assume that two effects contribute to the net resistive force: continual formation and breaking of cell-ECM attachments, and resistance to movement through viscous extracellular fluid. We assume that the resistance force associated with cell-ECM attachments has the same magnitude as limiting friction ${ℱ}^{\text{friction}}$, and acts in the opposite direction to the applied force $\left({\vec{𝐅}}^{\text{fric}}=-\frac{{\vec{𝐅}}^{\text{app}}}{\left|{\vec{𝐅}}^{\text{app}}\right|}{ℱ}^{\text{friction}}\right)$. The resistance force associated with cell movement through the extracellular fluid is modelled as a Stokes’ drag force; similar drag terms have been used in other ABMs [32–34,56,76].

Combining these forces, we write the classical equation of motion (4) as follows:


$$\begin{array}{c} \underset{\text{viscous limit}}{\underbrace{0=m\ddot{\vec{{𝐫}_{𝐢}}}}}={\vec{𝐅}}^{\text{app}}+{\vec{𝐅}}_{i}^{\text{drag}}+{\vec{𝐅}}_{i}^{\text{fric}} \end{array}$$ (4)

$$\begin{array}{c} \underset{\text{applied force}}{\underbrace{{\vec{𝐅}}_{i}^{\text{app}}=\sum_{\text{forces}}\vec{F_{i}}}}\;,\;\underset{\text{Stokes' drag}}{\underbrace{{\vec{𝐅}}_{i}^{\text{drag}}=-\nu \dot{\vec{r_{i}}}}}\;,\;\underset{\text{friction}}{\underbrace{{\vec{𝐅}}_{i}^{\text{fric}}=\left\{ \begin{array}{c} -{\vec{𝐅}}_{i}^{\text{app}}\text{ if }|{\vec{𝐅}}_{i}^{\text{app}}|<{ℱ}^{\text{friction}}\\ -\frac{{\vec{𝐅}}_{i}^{\text{app}}}{\left|{\vec{𝐅}}_{i}^{\text{app}}\right|}{ℱ}^{\text{friction}}\text{ otherwise} \end{array}\right.}} \end{array}$$ (5)

where ${\dot{\vec{𝐫}}}_{i}$ is the velocity of cell *i*, ν is the damping coefficient for Stokes’ drag, and $\sum {\vec{𝐅}}_{i}^{\text{app}}$ represents the total force acting on cell *i*, due to physical contact with its neighbours.

By neglecting inertial terms (over-damped, viscous limit), Equation (4) can be rearranged to give:


$$\begin{array}{c} {\dot{\vec{𝐫}}}_{i}=\left\{ \begin{array}{cc} \frac{1}{\nu }\left(\left|{\vec{𝐅}}_{i}^{\text{app}}\right|-{ℱ}^{\text{friction}}\right)\frac{{\vec{𝐅}}_{i}^{\text{app}}}{\left|{\vec{𝐅}}_{i}^{\text{app}}\right|} & \text{ if }\left|{\vec{𝐅}}_{i}^{\text{app}}\right|>{ℱ}^{\text{friction}}\\ 0 & \text{ otherwise } \end{array}\right. \end{array}$$ (6)

Equation (6) differs from the equation of motion typically used in node-based models: it allows cells to resist applied forces and remain static if the magnitude of the applied force ($\left|{\vec{𝐅}}^{\text{app}}\right|$) is less than the threshold value ${ℱ}^{\text{friction}}$. Equation (6) is solved at discrete timesteps using a forward Euler approach, noting that all simulations are halted before cells reach the boundary of the computational domain.

#### 2.1.5. Vessel Force Balance.

Vessel occlusion arises when proliferation of neighbouring tumour cells generates mechanical forces that compresses and occludes vessels. Similarly to tumour cells as described above, we use force balance to derive equations of motion for the vessel due to applied contact forces (details on these contact forces given in Section S1.3.1 in S1 Text). Different to tumour cells, vessels in our model do not move and instead we model the applied forces as acting on and deforming the vessel wall. We assume that vessel deformation is driven by the difference between the external solid pressure *P*_*i*_, and the internal vessel pressure $P^{⋆}$.

As mentioned above we model vessels by their circular cross-sections with dynamic radii, and we further assume that the mean force per unit length exerted by cells surrounding a blood vessel approximates the solid pressure *P*_*i*_ experienced by the vessel. Precisely, letting *N* be the number of cells neighbouring a given vessel and $\vec{F_{j}}$ be the force applied by neighbour j(j=1,…,N) to the vessel, we can write the pressure contribution $\rho_{j}$ from neighbour *j* as:


$$\begin{array}{c} \rho_{j}=\frac{1}{L_{0}}\vec{F_{j}}\cdot \frac{\vec{r_{i}}-\vec{r_{j}}}{\left|\vec{r_{i}}-\vec{r_{j}}\right|} \end{array}$$ (7)

where *L*_0_ is the length of the cell-vessel interface over which contact pressure is applied, $\vec{r_{i}}$ is the vessel’s position and $\vec{r_{j}}$ is the position of neighbour *j*. We approximate each cell-vessel interface to have length *L*_0_ = 1 since cell boundaries are poorly defined in the overlapping-spheres framework we are using. We also assume that each neighbour’s pressure contribution $\rho_{j}$ is evenly distributed along the vessel boundary and therefore assume that the pressure *P*_*i*_ applied to a vessel is the average of the pressure contributions from each neighbour:


$$\begin{array}{c} P_{i}=\frac{1}{N_{c}}\sum_{j}^{N_{c}}\underset{\rho_{j}}{\underbrace{\frac{1}{L_{0}}\vec{F_{j}}\cdot \frac{\vec{r_{i}}-\vec{r_{j}}}{\left|\vec{r_{i}}-\vec{r_{j}}\right|}}} \end{array}$$ (8)

Equation (8) defines how we model the mechanical pressure experienced by a vessel due to intercellular forces. We acknowledge that alternative methods could be used to approximate mechanical pressure, but consider this simple functional form to be appropriate here given uncertainties in how the modelled spring forces translate into physical contact forces.

The difference between the external pressure *P*_*i*_ (8) and internal pressure $P^{⋆}$ generates a force of magnitude $L_{0}\left(P_{i}-P^{⋆}\right)$ acting to occlude ($P_{i}>P^{⋆}$) or dilate ($P_{i}<P^{⋆}$) the vessel. By balancing this against a damping force with damping coefficinet $\nu_{r}$ and assuming that the maximum vessel radius is ${\hat{R}}_{\text{max}}$, we derive an equation of motion for the vessel’s radius $R_{i}=R_{i}\left(t\right)$:


$$\begin{array}{c} \nu_{r}\frac{dR^{⋆}}{dt}=-L_{0}\left(P-P^{⋆}\right)ℋ\left({\hat{R}}_{\text{max}}-R^{⋆}\right),\\ \text{where}\;ℋ\left(x\right)=\left\{ \begin{array}{c} 1\text{ if }x>0,\\ 0\text{ otherwise.} \end{array}\right. \end{array}$$ (9)

#### 2.1.6. Radiotherapy.

Our model generates heterogeneous oxygen landscapes due to interactions between the tumour cells and the surrounding vasculature. We demonstrate the impact of this heterogeneity by simulating tumour responses to radiotherapy using a simple model of cell death following exposure to radiotherapy. We focus on the cell killing due to radiotherapy as it is known to be sensitive to oxygen levels [65,66].

We adapt a simple Linear-Quadratic (LQ) model [62] to describe the probability that a tumour cell dies following exposure to a single dose of radiotherapy, P(death|dose). This probability depends on the radiotherapy dose, the tumour’s oxygen status and the local oxygen concentration:


P(death|dose)=1−e−γ(α·OMF·dose+β(OMF·dose)2)α=0.3Gy−1,β=0.03Gy−1,γ={0.25 if Hypoxic,1 otherwise. (10)

γ accounts for reduced radio-sensitivity of quiescent, hypoxic cells due to their lack of DNA replication [61], whilst the Oxygen Modification Factor (**OMF**) accounts for reduced effectiveness of radiotherapy due to the lack of oxygen needed to generate DNA damaging free radicals [63]:


$$\begin{array}{c} \text{OMF}=\left\{ \begin{array}{cc} \frac{1}{3} & \text{ if }\omega <0.1,\\ 1 & \text{ otherwise,} \end{array}\right. \end{array}$$ (11)

where ω is the local oxygen concentration.

In radiotherapy simulations, treatment is applied at *t* = 21 days and simulations continued for a further 21 days. Any cells killed by radiotherapy are labelled as ‘apoptotic’ and die in the same way as necrotic cells (for further details, see Section S1.1.3 in S1 Text).

### 2.2. Simulating tumours

Each simulation starts with 4 tumour cells seeded at the centre of a 1mm by 1mm square domain with 400 ‘healthy’ blood vessels placed randomly without overlap. We initialise 600 additional locations at which new vessels can grow via angiogenesis; we term them ‘Pruned’ vessels (see Section 2.1.1). The randomness in the initial vessel distribution is distinct from other stochastic parts of the model and can be varied using a configuration seed parameter which is separate from the random seed used in the rest of the model. This allows stochastic simulations to be conducted in the same vascular environment if desired. Synthetic tumours grow for 42 days of simulation time or until the number of tumour cells exceeds 10,000. Snapshots of the tumour are saved at 10 hour timesteps.

We analyse the model’s qualitative behaviour by performing four parameter sweeps: two focused parameter sweeps in the absence of radiotherapy, one in the presence of radiotherapy, and an extensive Latin hypercube sampled parameter sweep in the absence of radiotherapy which we use to test the robustness of findings from the focused parameter sweeps. The parameter sweeps are summarised in Table 1.

**Table 1:** Summary of parameter sweeps.

Parameter Sweep	ℛ	Parameters Varied	*N*_*p*_	*N*_*c*_	*N*_*r*_	Total	Notes
$P^{⋆}-{ℱ}^{\text{friction}}$	No	${ℱ}^{\text{friction}}$, $P^{⋆}$	110	4	4	**1,760**	S2.1.1.
$\omega_{h}-P^{⋆}$	No	$\omega_{h}$, $P^{⋆}$	70	4	4	**1,120**	S2.1.2.
Radiotherapy	Yes	$P^{⋆}$, $\omega_{\text{angio}}$	100	4	4	**1,600**	S2.2.
Latin Hypercube	No	κ, $\omega_{h}$, $P^{⋆}$, ${ℱ}^{\text{friction}}$, η, ${\hat{R}}_{\text{occ}}$, ν, μ	5000	4	1	**20,000**	S2.1.3.
**Total**						**24,480**	

Table summarising parameter sweeps performed to analyse model.

ℛ: Whether radiotherapy was applied at day 21

*N*_*p*_: Number of parameter sets in parameter sweep

*N*_*c*_: Number of initial vessel configurations simulated

*N*_*r*_: Number of stochastic repetitions

Total: Total number of simulations in parameter sweep

### 2.3. Spatial analysis

#### 2.3.1. Tumour-vessel pair correlation function.

**Fig 2:**
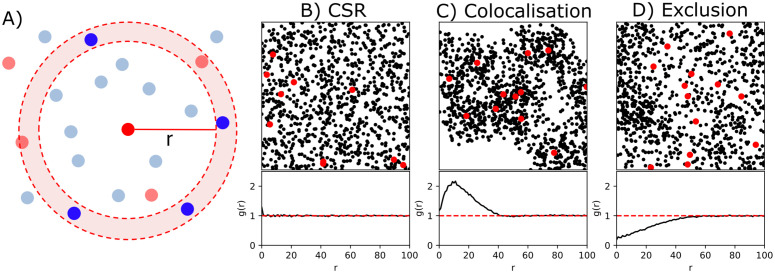
Schematic of cross pair correlation function. (A) Schematic demonstrating how the cross-PCF is calculated by drawing annuli around red points and counting the number of blue points which fall within the annuli. (B, C, D) Illustrations of the cross-PCF applied to synthetic datasets. Letting red points represent vessels and black points represent tumour cells, we demonstrate three basic spatial signatures. (B) Complete spatial randomness (CSR). The cross-PCF is flat ($g_{BT}(r)≃1$). (C) Colocalisation of vessels and tumour cells. The cross-PCF has a peak whose height indicates the strength of colocalisation and whose *r* value indicates the range of interaction distances at which the two agent types are positively correlated. (D) Negative correlation between vessels and tumour cell location. Here, *g*_*BT*_ < 1 for *r* < 50, indicating exclusion of tumour and vessels points at this length scale.

We use the cross pair correlation function (cross-PCF) to quantify the spatial distribution of blood vessels as they are remodelled by the tumour [71,72,77]. The tumour-vessel cross-PCF ($g_{BT}\left(r\right)$) measures the number of observed tumour-vessel pairs separated by distance *r*, relative to the number of such pairs expected under a null hypothesis of complete spatial randomness (CSR). We define annuli with inner radii *r* and outer radii *r* + *dr*, centred around each blood vessel, and consider how many tumour cells fall within these annuli (see Fig 2A). Letting $A_{r}\left(\vec{𝐱}\right)$ be the area of the intersection of an annulus of radius *r* centred at $\vec{𝐱}$ and the simulation domain, and defining an indicator function


$$\begin{array}{c} I_{\left[a,b\right)}\left(r\right)=\left\{ \begin{array}{cc} 1 & \text{ if }r\in \left[a,b\right),\\ 0 & \text{ otherwise,} \end{array}\right. \end{array}$$ (12)

the tumour-vessel PCF is defined as


$$\begin{array}{c} g_{BT}(r)=\frac{1}{N_{B}}\underset{i\in \{BV\}}{\sum }\underset{j\in \{T\}}{\sum }\left(I_{\left[0,dr\right)}\left(\left|\vec{x_{i}}-\vec{x_{j}}\right|-r\right)\underset{\text{normalization}}{\underbrace{{\left[\frac{N_{T}}{A}A_{r}\left(\vec{x_{i}}\right)\right]}^{-1}}}\right), \end{array}$$ (13)

where {*BV*} are blood vessels, {*T*} are tumour cells, *N*_*B*_ and *N*_*T*_ are the numbers of blood vessels and tumour cells respectively. Here, $g_{BT}(r)>1$ represents colocalisation of tumour cells and blood vessels at radius *r*, whereas $g_{BT}(r)<1$ indicates exclusion. Practically, the cross-PCF defined in Equation 13 is calculated at a discrete series of radii *r*_*k*_ such that *r*_0_ = 0, $r_{k+1}=r_{k}+dr$. We fix *dr* = 1 cell diameter and consider radii up to a maximum of *r* = 100 cell diameters.

To aid interpretation of the cross-PCF, Figs 2B,C,D show three synthetic datasets, involving red (representing vessels) and black (representing tumour cells) points, and their cross-PCF signatures. Under complete spatial randomness (CSR), with no spatial correlation between red and black points (Fig 2B), the cross-PCF is flat and close to 1 for all *r*. When black points cluster around red points (Fig 2C), there is a peak in the cross-PCF; its height (g(10)≃2.2) quantifies the increase in pairs observed separated by this distance compared to the number expected under CSR, and its location (r≃10) indicates the length scale at which clustering is strongest. Such cross-PCF signatures indicate increased tumour-vessel colocalisation at short distances which we interpret as the presence of vessels within the main tumour mass. In Fig 2D, black points are sparse around red points, and the resultant PCF has *g*(*r*) < 1 for *r* < 50. Such cross-PCF signatures indicate exclusion of blood vessels from the tumour.

The cross-PCF for small *r* will be important in our analysis because it indicates the local co-localisation, or exclusion, of blood vessels and tumour cells. Therefore, we define the mean cross-PCF value for 0<r≤5 cell diameters, denoted ${\bar{g}}_{BT}$, as:


$$\begin{array}{c} {\bar{g}}_{BT}=\frac{1}{5}\int_{0}^{5}g_{BT}\left(r\right)dr. \end{array}$$ (14)

#### 2.3.2. Tumour roundness.

To quantify a simulated tumour’s morphology, we apply a simple metric which describes the tumour’s roundness. First, we determine the tumour’s bounding polygon by calculating its α-shape [78] with critical parameter $\alpha =\frac{1}{2\text{ cell diameters}}$. The roundness of this polygon is defined as:


$$\begin{array}{c} \text{roundness}=\frac{4\pi \left(\text{area}\right)}{{\left(\text{perimeter}\right)}^{2}}, \end{array}$$ (15)

which is 1 for a circle and tends to 0 as the elongation of an oval goes towards infinity. While roundness could be quantified in other ways [79], including eccentricity and roughness [80], we use this metric because it is simple and interpretable, and captures changes in tumour morphology which provide insight into tumour behaviour.

### 2.4. Response to radiotherapy

The radiotherapy model described in Section 2.1.4 determines which cells are killed. To quantify its effects, we use two metrics. First, we measure χ, the percentage of tumour cells that die during a simulated dose of radiation. Secondly, we record *T*_*R*_, the time it takes for the tumour to regrow to the same number of cells as immediately before the radiation dose was applied. Denoting by $N_{R}^{\prime }$ and *N*_*R*_ the number of cells killed during and the number of live cells immediately before radiotherapy respectively, we define $\chi =\frac{N_{R}^{\prime }}{N_{R}}$. The recovery time, *T*_*R*_, is the time taken for the number of tumour cells to return to *N*_*R*_. As stated in Section 2.1.4, simulations are continued for 21 simulated days after treatment, by which time all tumours had regrown to at least *N*_*R*_ cells.

To understand the behaviour of *T*_*R*_ it is useful to study how the tissue’s vasculature evolves before and after radiotherapy. Particularly relevant is Ω, the oxygen capacity of the vasculature, which is defined as follows:


$$\begin{array}{c} \Omega =\sum_{i\in \text{\{BV\}}}\alpha_{i}, \end{array}$$ (16)

where {BV} are blood vessels, and the scaling factor $\alpha_{i}$ determines vessel *i*’s ability to supply oxygen as defined in Equation (2).

## 3. Results

In this section, we show that including a friction force in our off-lattice ABM enables pressure to accumulate within a tumour. We then investigate how pressure accumulation impacts blood vessel occlusion, and show how dynamic interactions between blood vessels and tumour cells affect a tumour’s growth dynamics and its morphology. Finally, we show how vascular remodelling impacts tumour responses to radiotherapy.

### 3.1. Simulating a friction force enables accumulation of pressure

**Fig 3:**
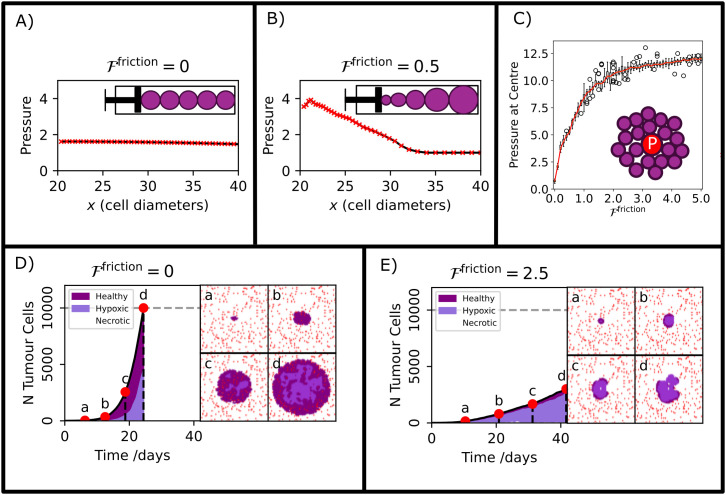
Friction drives pressure accumulation inside tumours. (A) 1D simulation in which a chain of cells is compressed by slowly moving the left boundary from *x* = 0 to *x* = 20, in the absence of friction (${ℱ}^{\text{friction}}=0.0$). At equilibrium, all cells experience the same pressure. (B) The simulation in A) is repeated with $F^{friction}=0.5$ (all other parameters held fixed). At equilibrium, cells closer to the applied force experience higher pressure than those farther away. In contrast to A), the force has no impact on cells further than 34 cell diameters from the applied force. (C) Tumour cells surrounding a single blood vessel grow until the tumour reaches an equilibrium size. The equilibrium pressure on the blood vessel, averaged over 16 repeats, is plotted as a function of the friction strength, demonstrating that without friction, pressure does not accumulate in the centre of the cluster. (D,E) Tumour growth dynamics exhibited by our model in the absence (D) and presence (E) of a friction force, with all other model parameters fixed at the default values specified in Table A in S1 Text. Snapshots a-d show the simulation at four highlighted timepoints; red: blood vessels, purple: healthy tumour, lilac: hypoxic tumour, white: necrotic tumour. (D) Simulation without friction (${ℱ}^{\text{friction}}=0$), (E) Simulation including friction (${ℱ}^{\text{friction}}=2.5$). Without friction, blood vessels withstand the pressure due to tumour growth, resulting in high tissue oxygenation and a large, rapidly growing tumour. When friction is included, pressure accumulates causing blood vessel occlusion, the formation of a hypoxic core inside the tumour, and slower tumour growth.

We first consider a simple model in which a compressive force is applied at the left hand boundary of a 1D chain of 100 cells. Specific details of this toy model are given in Section S1.4 in S1 Text. We compare the pressure that accumulates when there is no friction force (Fig 3A), and when a friction force is included (Fig 3B).

The pressure distributions plotted in Figs 3A and B show that incorporating friction permits the accumulation of pressure within the chain of cells. In the absence of friction (Fig 3A), the compressive force is transmitted evenly to all cells in the chain, creating constant pressure. When friction is included (Fig 3B), pressure accumulates within cells which are close to the compressed boundary on the left, and decreases with distance from the boundary, eventually reaching a constant value, so that distant cells do not move in response to the applied force.

In a second example, the growth of a population of tumour cells is supported by a single blood vessel which is unaffected by pressure and, as such, cannot be occluded. In this case, the tumour grows around the vessel until its reaches a stable, equilibrium size. Fig 3C shows how the value of the friction strength ${ℱ}^{\text{friction}}$ affects the compressive pressure acting on the blood vessel at the centre of the tumour when it reaches its equilibrium size. The pressure that accumulates at the centre of the tumour is an increasing, saturating function of ${ℱ}^{\text{friction}}$, demonstrating that the inclusion of friction causes the pressure associated with tumour growth to accumulate.

These simple examples illustrate how frictional forces may impact the distribution of pressure in a simulated tissue, and in Figs 3D,E we show that this can impact tumour growth dynamics. Figs 3D,E show typical simulations in which a tumour is seeded in a vascular environment, in the absence (D) and presence (E) of a friction force. In Fig 3D, we fix ${ℱ}^{\text{friction}}=0$. The pressure disperses uniformly across the cells and does not accumulate inside the tumour. As a result, the blood vessels remain functional and a large tumour, containing approximately 10,000 cells, forms after approximately 23 days. By contrast, Fig 3E shows that when ${ℱ}^{friction}>0$ (all other model parameters held fixed), pressure accumulates inside the tumour, vessels become occluded, leading to the formation of a hypoxic core and slowing tumour growth.

### 3.2. Pressure accumulation causes vessel occlusion and formation of avascular tumours

We use the tumour-vessel cross-PCF, $g_{BT}\left(r\right)$, to quantify the spatial distribution of blood vessels within a tumour and, in so doing, evaluate the degree of vessel remodelling.

**Fig 4:**
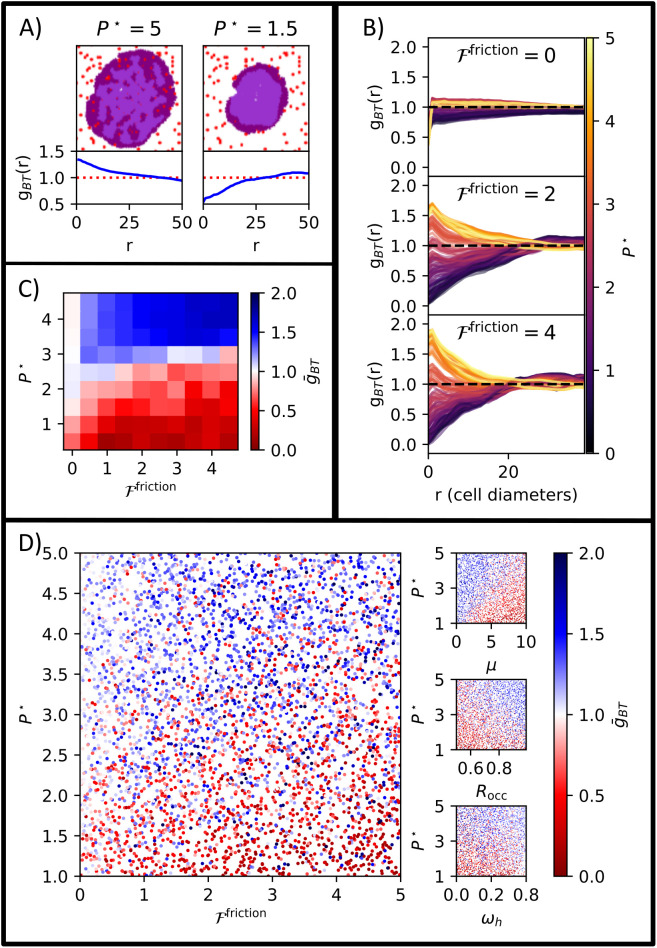
Pressure accumulation drives vessel occlusion and emergence of avascular tumours. (A) Tumour distributions and tumour-vessel cross-PCFs, *g*_*BT*_(*r*), from two representative simulations with different values of $P^{⋆}$ (*t* = 28 days). Left: High $P^{⋆}=5$ leads to a well-vascularised tumour, with colocalisation of tumour cells and vessels ($g_{BT}>1$ for *r* < 25). Right: Low $P^{⋆}=1.5$ leads *t*o the formation of an avascular tumour, which lacks vessels due to occlusion (*g*_*BT*_ < 1 for *r* < 25). Red: blood vessels, purple: normoxic tumour, lilac: hypoxic tumour. For this example ${ℱ}^{\text{friction}}=1$ and other parameters are default values given in Table A in S1 Text. (B) Tumour-Vessel cross-PCF signatures for fixed ${ℱ}^{\text{friction}}$ coloured by $P^{⋆}$ (see Section S2.1.1 in S2 Text), demonstrating that, when ${ℱ}^{\text{friction}}>0$, the value of $P^{⋆}$ can substantially impact tumour vascularisation (characterised by *g*_*BT*_ signatures that are qualitatively similar to those for the vascular and avascular tumours shown in (A). For ${ℱ}^{\text{friction}}=0$, all tumours are well-vascularised, for all values of $P^{⋆}$. For ${ℱ}^{\text{friction}}>0$, small values of $P^{⋆}$ lead to formation of avascular tumours and larger $P^{⋆}$ values lead to well-vascularised tumours. (C) ${\bar{g}}_{BT}$ (Section 2.3.1), a metric measuring the low (red) or high (blue) vascularisation of the tumour across the $P^{⋆}-{ℱ}^{\text{friction}}$ 2-parameter sweep (see S2 Text). There is a threshold value of $P^{⋆}$, which depends on ${ℱ}^{\text{friction}}$, below which tumours become avascular. (D) 2-parameter projections of ${\bar{g}}_{BT}$ across a multidimensional Latin hypercube parameter sweep (Section S2.1.3 in S2 Text). Main panel shows ${\bar{g}}_{BT}$ as a function of $P^{⋆}$ and ${ℱ}^{\text{friction}}$, and shows a similar trend to panel C but with some noise due to changes in other model parameters. Sub panels show how, in the same simulations, ${\bar{g}}_{BT}$ varies with three additional parameters; cell stiffness μ, vessel occlusion ${\hat{R}}_{\text{occ}}$ and tumour hypoxia sensitivity $\omega_{h}$. The values of these parameters impact ${\bar{g}}_{BT}$, suggesting they contribute to whether a tumour remains vascular.

Fig 4A shows how $g_{BT}\left(r\right)$ can be used to distinguish vascular and avascular tumours. We consider two simulations, generated using the same parameter values, except for $P^{⋆}$ (high $P^{⋆}$ left, low $P^{⋆}$ right), and show the final simulation timepoints alongside the corresponding tumour-vessel cross-PCFs. For high $P^{⋆}$, the blood vessels do not experience sufficient pressure to be occluded, and, instead, dense, well vascularised cell clusters surround them. The colocalisation of tumour cells and blood vessels is captured in the cross-PCF, with $g_{BT}\left(r\right)>1$ for *r* < 25. Conversely, for low $P^{⋆}$, the accumulated pressure exceeds the vessel pressure, causing vessel occlusion and the formation of an avascular tumour. The absence (or exclusion) of vessels from the tumour is captured in the cross-PCF, with *g*_*BT*_ < 1 for *r* < 25.

Fig 4B shows the *g*_*BT*_(*r*) signature associated with the final simulation timepoints from a 2-parameter sweep over ${ℱ}^{\text{friction}}$ and $P^{⋆}$ (see S2.1.1 in S2 Text). When ${ℱ}^{\text{friction}}>0$ (middle and bottom plots of Fig 4B) the cross-PCFs are heterogenous, and qualitative comparison with the *g*_*BT*_ signatures in 4A indicates that the tumours range from well vascularised to avascular. When ${ℱ}^{\text{friction}}>0$, the value of $P^{⋆}$ determines how the *g*_*BT*_ signatures transition from those associated with well vascularised tumours in which cells cluster around vessels to those associated with avascular tumours in which vessels have been occluded by tumour growth. In contrast, when ${ℱ}^{\text{friction}}=0$ (uppermost panel of Fig 4B), the cross-PCF signatures remain relatively flat for all values of $P^{⋆}$. In these cases, the tumour grows alongside the vessels with pressure quickly dissipating as cells are not anchored to the substrate. This both restricts the formation of dense clusters around vessels and prevents vessel occlusion, resulting in flatter *g*_*BT*_ signatures. These results highlight the importance of friction in our model: ${ℱ}^{\text{friction}}$ allows cells to anchor to the substrate and prevents the dispersion of local mitotic forces, which drives the accumulation of pressure. The magnitude of the accumulated pressure relative to $P^{⋆}$ determines whether vessels become occluded, and explains why the dependence of vascularisation (as characterised by *g*_*BT*_) on $P^{⋆}$ disappears when ${ℱ}^{\text{friction}}=0$; pressure cannot accumulate when ${ℱ}^{\text{friction}}=0$.

Using the metric ${\bar{g}}_{BT}$ to quantify vascularisation as ${ℱ}^{\text{friction}}$ and $P^{⋆}$ vary reinforces this interpretation. The results in Fig 4C show that low values of $P^{⋆}$ result in ${\bar{g}}_{BT}<1$ (avascular tumours), whilst high values result in ${\bar{g}}_{BT}>1$ (vascular tumours). The transition between ${\bar{g}}_{BT}>1$ and ${\bar{g}}_{BT}<1$ is pronounced when ${ℱ}^{\text{friction}}>0$ and becomes less pronounced as ${ℱ}^{\text{friction}}\to 0$. We note further from Fig 4C that ${ℱ}^{\text{friction}}$ defines the threshold value of $P^{⋆}$ at which a tumour switches from being vascular to avascular.

In order to determine the robustness of the trends seen in this 2-parameter sweep, we performed a larger, Latin-hypercube parameter sweep, varying 8 parameters (Section S2.1.3 in S2 Text). Fig 4D shows a projection of the Latin hypercube parameter sweep in which $P^{⋆}$ and ${ℱ}^{\text{friction}}$ range over the same values as in panel C. We observe the same qualitative trends, with reduced $P^{⋆}$ resulting in vessel occlusion, and the value of ${ℱ}^{\text{friction}}$ determining the $P^{⋆}$ threshold at which this occurs. However the relationship is noisier, indicating that other model parameters influence vessel exclusion.

Analysis of alternative projections in our Latin hypercube parameter sweep reveal 3 additional parameters which impact vessel exclusion (see sub panels in Fig 4D). These parameters relate to a cell’s ability to generate mechanical forces which can occlude vessels (μ), a blood vessel’s resistance to remodelling (${\hat{R}}_{\text{occ}}$) and a tumour cell’s oxygen requirements ($\omega_{h}$).

### 3.3. The oxygen landscape affects tumour morphology

In Section 3.2 we saw how the value of the vessel pressure $P^{⋆}$ determines whether a tumour’s vessels are occluded. Since blood vessels supply oxygen, varying $P^{⋆}$ also alters the oxygen landscape. In this section, we perform a 2-parameter sweep (Sect 2.1.2 in S2 Text) to determine how varying $P^{⋆}$ and $\omega_{h}$ affects a tumour’s growth dynamics (recall that $\omega_{h}$ is the threshold oxygen concentration below which cells halt proliferation and become quiescent).

**Fig 5:**
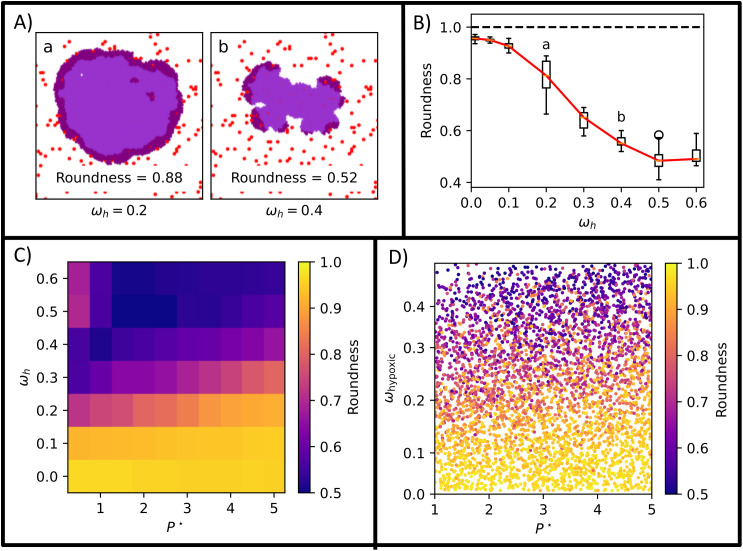
The oxygen landscape drives the formation of tumour lobes. Tumour roundness is calculated at the end timepoint for each simulation. (A) Representative tumours at *t* = 42 days from the $P^{⋆}-\omega_{h}$ parameter sweep (see Section S2.1.2 in S2 Text) showing how tumour morphology becomes more lobular as $\omega_{h}$ increases from $\omega_{h}=0.2$ (Left) to $\omega_{h}=0.4$ (Right), and how this can be captured by the roundness score. Red: blood vessel, purple: normoxic tumour, lilac: hypoxic tumour. (B) Effect of varying $\omega_{h}$ on tumour roundness when $P^{⋆}=2$, showing a sigmoidal relationship between $\omega_{h}$ (oxygen requirement) and the extent to which the tumour forms lobes. Markers a and b indicate the parameter values for the tumours shown in panel A. (C) Heatmap showing how roundness changes as $P^{⋆}$ and $\omega_{h}$ vary. Roundness scores are averaged across 4 realisations for each parameter set and show that smaller values of $P^{⋆}$ shift the sigmoidal relationship in panel B towards smaller values of $\omega_{h}$. (D) 2-dimensional projection from our multidimensional Latin hypercube sweep (Section S2.1.3 in S2 Text) onto the same axes as C, demonstrating that the behaviour in our 2-parameter sweep is robust to variations in other parameters in the multidimensional parameter sweep.

First, we show how changing $\omega_{h}$ can affect tumour size, composition and morphology at *t* = 42 days. In Fig 5A, the tumour on the left has a lower $\omega_{h}$ value. As a result, its cells can withstand lower oxygen concentrations and remain viable at greater distances from blood vessels, leading to a compact tumour mass. The tumour on the right has a higher $\omega_{h}$ value which limits its growth to small lobes that surround blood vessels.

We quantify tumour morphology using the roundness score (see Section 2.3.2), observing that roundness decreases as $\omega_{h}$ increases and the tumour becomes more lobular. Fig 5B shows how tumour roundness decreases in a sigmoidal manner as $\omega_{h}$ increases, indicating that as oxygen demand increases, the tumour forms lobes and becomes less round. Fig 5C shows how tumour roundness changes as $P^{⋆}$ and $\omega_{h}$ vary. As in Fig 5B, the roundness score decreases as $\omega_{\text{h}}$ increases. Varying $P^{⋆}$ shifts the sigmoid-like dependence on $\omega_{h}$ in Fig 5B: for smaller $P^{⋆}$, the roundness score decreases at smaller $\omega_{h}$. The observed change in roundness can be understood as the tumour being restricted to lobes that surround blood vessels when the oxygen supply (low $P^{⋆}$) is insufficient for the oxygen requirement ($\omega_{h}$). We performed similar analyses to investigate how tumour mass and hypoxic fraction vary across parameter space. As expected, increased vascularisation resulted in larger and better oxygenated tumours (see S3 Text for details).

As before, we performed a larger Latin hypercube parameter sweep (Section S2.1.3 in S2 Text) to determine whether the trends for the roundness score persist when multiple model parameters vary. In Fig 5D we project results from the larger parameter sweep onto ($P^{⋆},\omega_{h}$) parameter space. The trends from Fig 5C persist, suggesting that the changes in tumour morphology described above are more sensitive to $P^{⋆}$ and $\omega_{h}$ than other parameters in the Latin hypercube (S2 Text).

### 3.4. Vascular remodelling impacts tumour response to radiotherapy

In this section we use our model to investigate how vessel remodelling impacts a tumour’s response to radiotherapy. We employ the simplified oxygen-dependent radiotherapy model and radiotherapy protocol described in Section 2.1.4: a single dose of radiotherapy is applied at *t* = 21 days after which the tumour grows for another 21 days. All parameters are fixed at default values (Table A in S1 Text), except for the vessel parameters $P^{⋆}$ and $\omega_{\text{angio}}$ which vary as described in Section S2.2 in S2 Text.

**Fig 6:**
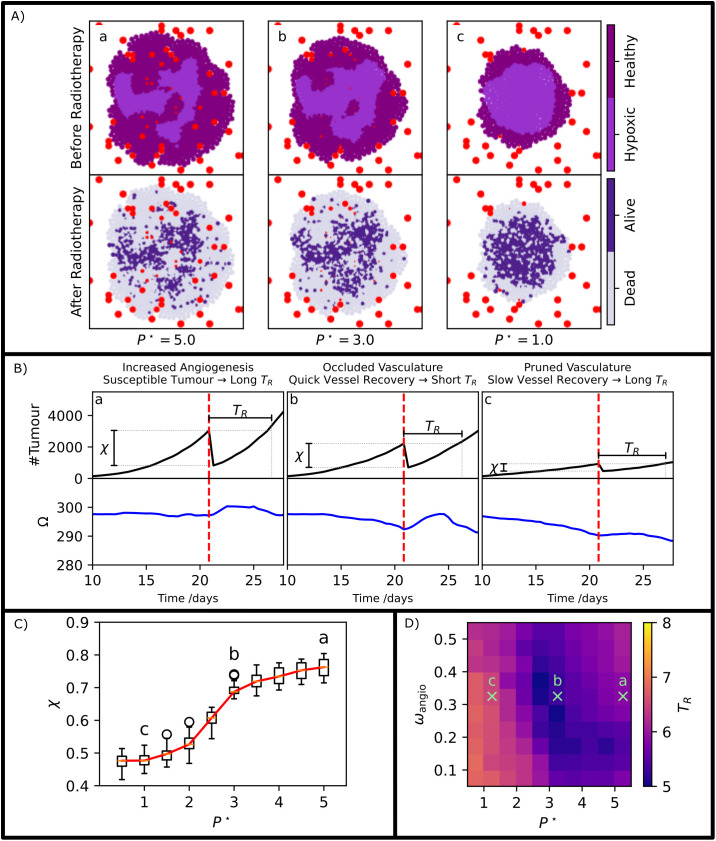
Effect of dynamic vasculature on radiotherapy outcomes. (A) Representative simulations showing tumours immediately before and after exposure to radiotherapy for different values of $P^{⋆}$. Large values of $P^{⋆}$ ($P^{⋆}=5.0$ in ‘a’) give rise to well oxygenated tumour which are extremely sensitive to radiotherapy so that a large proportion of the tumour cells are killed by radiotherapy. As $P^{⋆}$ decreases ($P^{⋆}=3.0$ in ‘b’ and $P^{⋆}=1.0$ in ‘c’), the tumour forms a radio-resistant, hypoxic core which enables a larger proportion of tumour cells to survive treatment. For these tumours, $\omega_{\text{angio}}=0.3$ and other parameters are fixed at the default values given in Table A in S1 Text. (B) Tumour and blood vessel growth trajectories for the simulations shown in a. The percentage of tumour cells killed (χ) decreases as $P^{⋆}$ decreases, reinforcing the results from panel A where low $P^{⋆}$ creates radiotherapy resistance. The parameter Ω, defined in 2.4, measures the vasculature’s oxygen capacity. (C) Variation in χ as $P^{⋆}$ varies. At low $P^{⋆}$ vessel occlusion causes hypoxia and radiotherapy resistance. As $P^{⋆}$ increases, both vascularisation and tissue oxygenation increase, and we identify a threshold value of $P^{⋆}$ at which tumours switch from low to high oxygenation. (D) Change in the post-radiotherapy recovery time *T*_*R*_ as $P^{⋆}$ and $\omega_{\text{angio}}$ vary. A valley of fast recovery times (‘b’) extends from a region with low $P^{⋆}$ and high $\omega_{\text{angio}}$ to a region with high $P^{⋆}$ and low $\omega_{\text{angio}}$.

Fig 6A shows representative simulated tumours that were treated with radiotherapy on day 21. Parameters are given in Section S2.2 in S2 Text. The vasculature for Tumour (a) has a large value of $P^{⋆}$ and is, therefore, large, well oxygenated and sensitive to radiotherapy. Tumour (c) has a low value of $P^{⋆}$. As a result, its vessels are easily occluded and the oxygen supply is low, leading to the formation of a radio-resistant, hypoxic core. The value of $P^{⋆}$ for Tumour (b) and its response to radiotherapy are intermediate between those for Tumours (a) and (c). Whilst its immediate response to radiotherapy appears qualitatively similar to Tumour (a), its post radiotherapy growth is substantially different.

The growth trajectories of the tumour and vasculature for Tumours (a), (b) and (c) are plotted in Fig 6B. We use the percentage of tumour killed (χ) and post-radiotherapy recovery time (*T*_*R*_), described in Section 2.4, to quantify the tumour’s response to radiotherapy. We observe that χ decreases as $P^{⋆}$ decreases from Tumour (a) to (c), reinforcing our interpretation of Fig 6A above. Fig 6C shows how χ varies with $P^{⋆}$ across a slice of our parameter sweep (S2 Text) with $\omega_{\text{angio}}=0.3$. These results further support the relationship between χ and $P^{⋆}$ described above. Additionally, Fig 6C shows that there is a threshold $P^{⋆}$ ($P^{⋆}≃2.5$ for the chosen parameter regime) below which χ falls significantly due to the formation of a large hypoxic core. χ is strongly dependent on $P^{⋆}$, with $\omega_{\text{angio}}$ having a much smaller effect (for further details, see S3 Text).

Fig 6B suggests that the dependence of *T*_*R*_ on $P^{⋆}$ differs from that for χ, with *T*_*R*_ being smallest for Tumour (b). Whilst we might expect Tumour (a), with high $P^{⋆}$, to have a long *T*_*R*_ because it is highly radiosensitive, it is initially less clear why Tumour (c) also has a long recovery time. We can understand this by looking at the dynamics of Ω, the oxygen capacity of the vasculature (see Section 2.4). For Tumour (b), the vasculature recovers rapidly following radiotherapy, and *T*_*R*_ is short. For Tumour (c), the vasculature recovers more slowly, resulting in increased *T*_*R*_. This difference in vessel recovery depends on the condition of the vasculature pre-radiotherapy. For Tumour (b), $P^{⋆}$ is sufficiently small that vessels are readily occluded, causing hypoxia and conferring radiotherapy resistance. However, the value of $P^{⋆}$ is not small enough for vascular pruning. By contrast, for Tumour (c), $P^{⋆}$ is small enough for vascular pruning. The resulting hypoxia leads to radiotherapy resistance, and necessitates angiogenesis to replace pruned vessels post-radiotherapy, greatly slowing tumour recovery.

We investigate this behaviour further in Fig 6D, where we show how *T*_*R*_ changes as $P^{⋆}$ and $\omega_{\text{angio}}$ vary. We observe a valley of fast recovery times which includes Tumour (b) and extends from low $P^{⋆}$ and high $\omega_{angio}$ to high $P^{⋆}$ and low $\omega_{angio}$. In order to understand this landscape, we note that two competing effects contribute to the length of the recovery time *T*_*R*_. First, as $P^{⋆}$ and $\omega_{\text{angio}}$ increase, tumour cells are better oxygenated and, hence, more susceptible to radiotherapy, which causes χ to increase. As a result, *T*_*R*_ also increases because more tumour cells must proliferate to replace those removed by radiotherapy: this explains why Tumour (a) is characterised by high χ and *T*_*R*_. Secondly, for sufficiently small values of $P^{⋆}$ the vasculature is heavily pruned following radiotherapy. Here, even though the tumour is radiotherapy resistant due to poor oxygenation, the slow rate of vessel regrowth limits the overall rate of tumour regrowth, increasing the recovery time, *T*_*R*_. This explains why Tumour (c) has low χ and high *T*_*R*_. The valley of short recovery times forms where $P^{⋆}$ is low enough for poor oxygenation to confer radiotherapy resistance, but high enough that the vasculature is not pruned. Instead, the vasculature is occluded and, therefore, the death of tumour cells post-radiotherapy reduces the mechanical pressure on the vessels, allowing them to quickly recover and increase the supply of oxygen, enabling the tumour cells to regrow quickly. This explains why Tumour (b) recovers faster than Tumours (a) and (c).

## 4. Discussion

We have developed an off-lattice agent-based model that describes the growth of a population of tumour cells embedded within a vascular tissue. Our ABM accounts for mechanical interactions between the tumour cells and blood vessels and vascular remodelling. We used our model to study the effect that the dynamic oxygen landscape associated with vascular remodelling has on a tumour’s morphology and response to radiotherapy.

Our model accounts for the growth of new vessels stimulated by tumour hypoxia, as well as their occlusion and removal due to tissue stress caused by rapid tumour growth. The mechanical components of off-lattice ABMs typically operate in the non-inertial, viscous limit and introduce a resistive force (representing cell-substrate interactions) to balance external forces acting on individual cells. Most existing off-lattice ABMs model this resistive force via Stokes’ drag. In such models, pressure dissipates over the tissue domain due to the dependence of Stokes’ drag on the velocity. As a result, forces generated by proliferating cells are quickly transmitted to the tumour boundary, preventing tumour cells from generating sufficient mechanical pressure to occlude blood vessels. In order to enable vessel occlusion, we introduced into the mechanical model a friction force which accounts for the adhesive bonds that form between the cells and the extracellular matrix and prevents a cell from moving if the applied forces it experiences are below a threshold value. Our simulations show that including this friction force, together with Stokes’ drag, prevents rapid pressure dissipation and enables vessel occlusion due to mechanical pressure. We note that, as described, this force does not account for resistive forces at the boundary of a growing tumour from the surrounding tissue; we do not explicitly model these effects. Further studies are needed to explore whether constraints imposed by the surrounding tissue could be accounted for by varying the friction force applied to cells close to the tumour boundary (which may be approximated using alpha shapes, see Section 2.3.2).

In addition to vessel occlusion and pruning caused by rapid tumour growth, our vascular model accounts for the growth of new vessels in regions that experience prolonged hypoxia. We demonstrated that, in combination with friction, $P^{⋆}$ (the pressure within a vessel) determines how readily vessels are occluded and pruned from the tumour. In more detail, increased $P^{⋆}$ results in well vascularised tumours and decreased $P^{⋆}$ results in avascular tumours.

We showed further how changes in the vasculature may impact tumour morphology. We quantified how increased sensitivity of tumour cells to hypoxia (high $\omega_{h}$) can cause a tumour to form lobes that encircle blood vessels and become less round if there is insufficient oxygen supply (low $P^{⋆}$). In the supporting information, we demonstrate that changes to the vasculature may impact other tumour features, including its size and hypoxic fraction (S3 Text). These results illustrate the importance of considering vascular occlusion when modelling tumour growth. Our model also highlights the importance of accounting for friction in force-based models of tumour growth, in order to enable pressure to accumulate within the tissue and to occlude blood vessels.

Finally, we showed that the addition of friction and vessel remodelling can have a significant effect on tumour responses to radiotherapy. The vessel pressure parameter $P^{⋆}$ affects the percentage of the tumour killed by radiation, with high $P^{⋆}$ generating normoxic tumours that are susceptible to radiotherapy, and low $P^{⋆}$ resulting in hypoxic tumours which are radioresistant, supporting observations in earlier studies [58,64]. Tumours within which vessels have very high $P^{⋆}$ have increased recovery time. As $P^{⋆}$ decreases the recovery time decreases, matching the reduced sensitivity to radiotherapy. However, there is a threshold $P^{⋆}$ below which the vasculature becomes both severely occluded and, importantly, pruned. This leads to significantly increased recovery times, as tumour recovery is then limited by the slower process of vessel angiogenesis. These results highlight the substantial impact that vessel mechanisms can have on a tumour’s response to radiotherapy.

There are many ways in which we could extend the work presented in this paper. While our use of the Chaste computational framework would facilitate the extension of the tumour growth and oxygen diffusion aspects of our model to 3D, representing the vasculature in 3D presents considerable computational challenges. [37] and [39] offer two approaches for representing 3D vasculature, however both would require significant extensions to account for vessel occlusion and pruning.

Future work might relax our simplified view of the vasculature as a series of point sources of oxygen, instead modelling a dynamic network of connected blood vessels [41–48]. Microvessel CHASTE [81], a CHASTE module for studying blood flow through vascular networks, could be incorporated to more accurately capture the interplay between tumour growth and local haematocrit levels. It would be interesting to use this to study ‘collateral’ effects, in which occlusion of upstream vessels impacts downstream vessels.

In the future, it would also be interesting to incorporate a model of extracellular matrix (ECM) into our work [55,82–86], to explicitly capture the formation and breaking of cell–ECM adhesion bonds and to study their effects on cell migration and ECM deformation [87,88]. Active cell migration plays a key role in cancer invasion and is known to be influenced by solid stress [89]. Extending our model to investigate active cell migration into the surrounding ECM and stroma would therefore be of great interest. However, additional work is needed to better understand the underlying physics of cell motility, particularly the role of epithelial–mesenchymal transition in this process [90,91].

In future work it would be interesting to consider the impact that the extracellular fluid exerts on the mechanical pressure in the tumour [15,92,93]. When tumour cells are killed they disintegrate into extracellular fluid and residual cellular fragments are cleared by phagocytic immune cells, including macrophages. This process can generate high interstitial fluid pressure in the tumour core, which drives movement of extracellular fluid out of the tumour [49,53].

It would also be interesting to investigate the effect of vessel occlusion on other forms of therapy such as immunotherapy [94] and chemotherapy [95–97], which have been studied in similar vascular models focusing on angiogenesis [37,44,45]. The design principles used to develop our ABM mean that it is ideally suited to study the complex mechanisms involved in these treatments and their combinations.

In future work we could also compare our results with experimental data. Multiplex imaging methods [98] can locate and phenotype individual cell, paving the way for data that matches the spatial resolution of our model. The methods we have used to analyse our simulated data could be applied to such imaging data [68–70], and used for model parametrisation. Further development of such methods may enable validation of our model against experimental data, however suitable patient data is difficult to generate because our model is representative of small tumours at early stages of development whilst patient derived tumours are typically larger and well developed. Mechanisms neglected in the current model, such as immune infiltration, ECM remodelling and epithelial mesenchymal transition of cancer cells likely contribute to tumour morphology in such late stage tumours.

In summary, we have used our model to demonstrate that vessel remodelling, especially pressure-mediated vessel occlusion, can have a significant effect on both tumour growth and sensitivity to radiotherapy. In developing our model, we have also identified possible limitations of existing off-lattice models and shown how they can be resolved by incorporating a friction force, highlighting the need for more work to investigate how cell-substrate interactions can be incorporated into cell-based models of biological tissues, including vascular tumours.

## Supporting information

S1 Text Detailed Model Description. (PDF)

S2 Text Parameter Sweeps. (PDF)

S3 Text Additional Model Analysis. (PDF)

S1 Code Vascular remodelling. (GZ)
