# Supplementary material for: Exploring the relationship between vascular remodelling and tumour growth using agent-based modelling
Source: PLoS Comput Biol. 2026 May 15;22(5):e1012967. doi: 10.1371/journal.pcbi.1012967 (PMC13354106; doi:10.1371/journal.pcbi.1012967)
Supplement: S1 Text — (PDF) [file pcbi.1012967.s001.pdf]

S1 Detailed Model Description

Here we describe our off-lattice agent-based model of tumour and blood vessel coevolution. Our model is implemented using the Cancer, Heart And Soft Tissue Environment (Chaste) modelling package [S1–S3]. Chaste is an open-source, general purpose simulation package designed to solve computationally challenging, multiscale problems that arise in biology and physiology (see: `https://chaste.github.io`).

In our model framework, all tumour cells and blood vessels are represented by individual agents. Each agent has associated subroutines which govern its subcellular behaviour in the case of tumour cells, and vessel occlusion in the case of blood vessels. A mechanical module accounts for inter-agent forces which determine the movement of individual cell centres and the deformation of blood vessels (unlike cells, blood vessels are assumed to be static). An environment level PDE framework describes the spatio-temporal distribution of diffusible species such as oxygen.

Below, we start by describing the behaviours of the tumour cell agents; these are largely inherited from existing agent-based tumour models [S4]. We then describe the novel components of our model, which include dynamic remodelling of the blood vessel agents and the addition of a friction force in the equations of motion for the tumour cells.

S1.1 Tumour Cell Behaviour

A simple cell cycle component governs subcellular processes of tumour cells, primarily proliferation in response to environmental conditions.

S1.1.1 Oxygen Dependent Tumour Phenotype

In order to survive, cells consume oxygen which is supplied by blood vessel agents (see Section S1.2) that are distributed throughout the simulated tissue. The oxygen distribution is modelled using a reaction-diffusion PDE, with live cells as sinks and blood vessels as point sources of oxygen:

$$\frac{\partial \omega}{\partial t} = \underbrace{D_\omega \nabla^2 \omega}_{\text{Diffusion}} + \underbrace{\alpha\left(1-\omega\right)\sum_{i\in\text{blood vessels}} \alpha_i \delta\left(\vec{\mathbf{r}}-\vec{\mathbf{r_i}}\right)}_{\text{Diffusion from blood vessels}} - \underbrace{\kappa\omega \sum_{i\in\{\text{cells}\}} \delta\left(\vec{\mathbf{r}}-\vec{\mathbf{r_i}}\right)}_{\text{Cell consumption}} - \lambda\omega, \tag{1}$$

$$\frac{\partial \omega}{\partial n} = 0 \text{ on the domain boundary, where } n \text{ is the normal on the boundary,}$$

where D_ω and κ are the oxygen diffusion and cell consumption coefficients and $\vec{\mathbf{r_i}}$ is the location of agent i. We assume that the oxygen concentration in the blood vessels is constant, normalised to unity, and that the rate at which the i-th blood vessel supplies oxygen to the environment is proportional to the difference in oxygen concentration between the blood vessel and the tissue, and we denote by α the maximum rate of oxygen supply from a blood vessel. α_i is a scaling parameter defined by Equation (2) which describes how changes in the vessel radius R_i affect the oxygen supply rate:

$$\alpha_i = \frac{R_i - \hat{R}_{\text{min}}}{\hat{R}_{\text{max}} - \hat{R}_{\text{min}}} \tag{2}$$

where the parameters $\hat{R}_{\text{min}}$ and $\hat{R}_{\text{max}}$ define a vessel's minimum and maximum radius respectively. The evolution of a vessel's radius R_i is determined by balancing the forces that act between the vessel wall and neighbouring cells and is described in Section S1.3.3.

A tumour cell's local oxygen concentration determines its phenotype:

$$\text{Phenotype} = \begin{cases} \textbf{Normoxic} & \text{if } \omega > \omega_h \\ \textbf{Hypoxic} & \text{if } \omega_h \leq \omega < \omega_n \\ \textbf{Necrotic} & \text{if } \omega \leq \omega_n \end{cases} \tag{3}$$

where ω_h and ω_n are hypoxic and necrotic oxygen thresholds respectively.

S1.1.2 Tumour Cell Proliferation

In our model, tumour cells progress through their cell cycle at a rate which depends on their local oxygen concentration and cell size. Upon completing the cell cycle, they divide into two daughter cells. Cells can only progress through their cell-cycle if they are **Normoxic** and their area is larger than a proportion η of a prescribed target area. If these conditions are not met, then the cell-cycle is temporarily halted.

If ϕ_i represents a cell i's age or position in the cell cycle, then progress through the cell cycle can be represented as:

$$\frac{d\phi_i}{dt} = \mathcal{H}\left(\omega(\vec{\mathbf{r_i}}) - \omega_h\right)\mathcal{H}\left(\frac{R_i\left(t\right)^2}{\hat{R}_c^2} - \eta\right) \tag{4}$$

$$\mathcal{H}\left(x\right) = \begin{cases} 1 & \text{if } x \geq 0 \\ 0 & \text{if } x < 0 \end{cases}$$

where $R_i\left(t\right)$ and $\hat{R}_c$ represent a tumour cell's radius at time t and its target radius respectively. A cell's radius, $R_i\left(t\right)$, is the dynamic radius (calculated as half the average separation between cells whose radii are overlapping and therefore deemed to be physically contacting). The parameter $\hat{R}_c > 0$ represents a cell's equilibrium radius and is used to calculate the strength of intercellular forces between neighbouring cells (Section S1.3.1). With these definitions, $\frac{R_i(t)^2}{\hat{R}_c^2}$ represents the extent to which a cell is compressed, and we compare this quantity with a contact inhibition threshold η to determine whether a cell is contact inhibited from continuing through its cell cycle. We introduce this simple approximation for cell compression because cell boundaries are not well defined in our cell-centre-based model framework.

When a cell's age reaches its cell cycle duration (that is, when $\phi_i = \tau_i$), it divides into two daughter cells. One cell is located at the site of the parent cell, the other is placed at a distance of half a cell diameter away in a random direction. Both cells are reset with $\phi_i = 0$, and assigned new cell-cycle durations τ_i, chosen randomly from a uniform distribution:

$$\tau_i \sim U\left(0.75\overline{\tau}, 1.25\overline{\tau}\right) \tag{5}$$

with the parameter $\overline{\tau}$ representing the average cell cycle duration. The target radii of both daughter cells grow linearly from 0 to $\hat{R}_c$ over the course of an hour.

S1.1.3 Tumour Death

A cell with the **Necrotic** phenotype is considered dead. Its target radius shrinks linearly to 0 over τ_{apop} hours before it is removed from the simulation. While the radius is shrinking, the spring constant μ for any springs associated with the cell (described in Section S1.3.1) also shrinks to 0 to account for the cell's weakening structure. In this way, dying cells continue to occupy space and

physically interact with neighbouring cells, and they can be stably removed from the simulation once their size has decreased to zero (assumed to be through liquefaction).

S1.2 Blood Vessel Behaviour

In this section we explain how we model our blood vessel agents. Vessels are assumed to lie perpendicular to the plane and, therefore, we represent their cross-sections as circular agents whose radii evolve in response to forces applied by neighbouring cells. Below, we explain the different phenotypes that a vessel may adopt and the rules that determine phenotype transitions. These transitions describe vessel pruning and angiogenesis in our model. Later, we introduce the force balance that determines how a vessel's radius evolves over time, which describes occlusion due to applied forces (see Section S1.3.3).

S1.2.1 Vessel Phenotype

We distinguish three vessel phenotypes: 'Healthy', 'Occluded' and 'Pruned'. The size of a vessel's radius determines whether it is **Healthy** or **Occluded**:

$$\text{Vessel Phenotype} = \begin{cases} \textbf{Healthy} & \text{if } R_i >= \hat{R}_{\text{occ}} \\ \textbf{Occluded} & \text{if } R_i < \hat{R}_{\text{occ}} \end{cases} \tag{6}$$

where the parameter $\hat{R}_{\text{occ}}$ is the threshold vessel radius at which a vessel becomes occluded.

Both healthy and occluded vessels supply oxygen to the tissue, at a rate dependent on the vessel's radius (Section S1.1.1). We distinguish the **Occluded** phenotype as vessels which are sufficiently stressed that they are candidates to become **Pruned**. Vessels with the **Pruned** phenotype have been irreparably damaged, are considered dead and have effectively been removed from the simulation (see Section S1.2.2). **Pruned** vessels may transition to the **Healthy** phenotype following successful angiogenesis. The rules we use to model vessel pruning and angiogenesis are described below.

S1.2.2 Vessel Pruning

We assume that vessels are **Pruned** if they remain **Occluded** for longer than τ_{prune}, where $\tau_{\text{prune}} > 0$ is a fixed parameter. Denoting by $\gamma_i = \gamma_i(t)$ the amount of time for which vessel i has been occluded, we assume further that

$$\begin{cases} \gamma_i = 0 & \text{if } \textbf{Healthy} \\ \frac{d\gamma_i}{dt} = 1 & \text{if } \textbf{Occluded}, \end{cases} \tag{7}$$

and that vessel i switches to **Pruned** if $\gamma_i > \tau_{\text{prune}}$. The radius of **Pruned** vessels no longer evolves according to Equation 6 and they do not supply oxygen to the surrounding tissue. They return to the **Healthy** state if angiogenesis occurs.

S1.2.3 Vessel Angiogenesis

Vessel pruning reduces the oxygen supply to the domain. This is compensated for by angiogenesis, in which new blood vessels form. We incorporate angiogenesis into our model by viewing **Pruned** vessels as candidate sites for angiogenesis. **Pruned** vessels return to a **Healthy** phenotype if two conditions are met: First, there must be at least one **Healthy** vessel within distance D_{angio} from which the new vessels are assumed to emerge. Secondly, the candidate location must have experienced sustained hypoxia for an extended period, representing the time taken for local accumulation

of angiogenic factors and the formation of a new vessel from neighbouring, pre-existing vessels (sprouting outside of the simulation plane).

Accordingly, we monitor the hypoxic stress Γ_i at the location of **Pruned** vessel i and assume that $\Gamma_i = \Gamma_i(t)$ evolves as follows:

$$\begin{cases} \frac{d\Gamma_i}{dt} & = 1 \text{ if } \omega < \omega_{\text{angio}} \\ \Gamma_i & = 0 \text{ otherwise} \end{cases}, \tag{8}$$

where ω is the local oxygen concentration and the parameter ω_{angio} denotes the oxygen threshold below which angiogenic factors are produced by the tumour cells. The process of angiogenesis can then be described by the following pseudocode.

Algorithm A: Vessel Angiogenesis Algorithm. Rules which define when a vessel reverts from a **Pruned** to a **Healthy** phenotype, as a result of angiogenesis. In order for a vessel to undergo angiogenesis, it must be hypoxically stressed ($\omega < \omega_{\text{angio}}$) for longer than τ_{angio} and there must be a **Healthy** vessel within a distance R^{angio} from which new vessels can sprout.

```
Require: τ_angio ;                                   ▷ Angiogenesis delay
Require: D_angio ;                                   ▷ Maximum sprouting distance
Require: P_angio ;                                   ▷ Angiogenesis Probability
foreach Pruned vessel do
    Γ ← GetHypoxicStress (vessel)
    if Γ < τ_angio then
        continue
    D ← DistanceToClosestHealthyVessel()
    if D > D_angio then
        continue
    p ∼ Uniform [0, 1)
    if p < P_angio then
        Regrow(vessel)
end
```

S1.3 Force Balance

In our ABM, cells and blood vessels are represented by the positions of their centres and their radii. Agents whose centres are within an interaction distance of $3\hat{R}_c$ are defined as neighbours and may exert contact forces on each other. In the next section we describe the functional forms used to model the contact forces, before explaining how force balances are used to determine how cells move and how blood vessels deform in response to these applied contact forces.

S1.3.1 Inter-Agent Contact Forces

As noted above, agents whose centres are separated by a distance less than $3\hat{R}_c$ are deemed neighbours and may exert contact forces on each other. The centres of neighbouring cells are connected with non-linear springs which can generate repulsive and attractive forces, modelling volume exclusion and intercellular adhesion respectively Since blood vessels do not move, we assume that the

contact forces they experience act on the vessel wall and regulate the size of their radii. To remain within our framework, where non-linear springs connect the centres of neighbouring agents, we implement different functional forms for cell-cell and cell-vessel contact forces (by design, the fixed locations of all vessel centres are separated by distances of at least $3\hat{R}_c$ so that vessel-vessel contact forces can be ignored).

Cell-Cell Interaction Force

Suppose that cells i and j are neighbours, whose centres are connected by a non-linear spring, whose natural spring length L_{ij} corresponds to the sum of their target equilibrium radii (R_i and R_j respectively). Following [S4] and [S5], we assume that the force $\vec{\mathbf{F}}_{ij}^{cc}$ exerted on cell i by cell j is such that:

$$\left|\vec{\mathbf{F}}_{ij}^{cc}\right| = \begin{cases} \mu L_{ij} \ln\left(1 + \frac{\Delta x_{ij}}{L_{ij}}\right) & \text{if} \quad \Delta x_{ij} \leq 0, \\ \mu L_{ij} \Delta x_{ij} \exp\left(-\delta \frac{\Delta x_{ij}}{L_{ij}}\right) & \text{if} \quad \Delta x_{ij} > 0, \end{cases} \tag{9}$$

where $L_{ij} = R_i + R_j$ is the equilibrium distance between the centres of cells i and j, $\Delta x_{ij} = |\vec{\mathbf{r_i}} - \vec{\mathbf{r_j}}| - L_{ij}$ is the current displacement from this equilibrium distance, the parameter μ determines the spring stiffness, and δ determines the strength of intercellular adhesion. We assume further that the force acts in the direction of the vector connecting the two cell centres, and is attractive if $\Delta x_{ij} > 0$ and repulsive otherwise. By Newton's Third Law, the force exerted on cell i by cell j is equal and opposite to that applied on cell j by cell i ($\vec{\mathbf{F}}_{ij}^{cc} = -\vec{\mathbf{F}}_{ji}^{cc}$).

Since all cells in our model, apart from necrotic or newly-divided cells, have a desired radius of $\hat{R}_c$, in most cases the equilibrium distance $L_{ij} = 2\hat{R}_c$. Exceptionally, the target cell radius $\hat{R}_c$ of necrotic and newborn cells evolve according to the rules described in Sections S1.1.2 and S1.1.3.

The functional form of Equation 9 is such that $\left|\vec{\mathbf{F}}_{ij}^{cc}\right| \approx 0$ when $|\vec{\mathbf{r_i}} - \vec{\mathbf{r_j}}| \approx 3R_t$, the maximum distance at which we assume cells can interact. When $\Delta x_{ij} = 0$, $\left|\vec{\mathbf{F}}_{ij}^{cc}\right| = 0$, and the contact force becomes strongly repulsive as the distance between the cell centres decreases ($\left|\vec{\mathbf{F}}_{ij}^{cc}\right| \to \infty$ as $|\vec{\mathbf{r_i}} - \vec{\mathbf{r_j}}| \to 0$).

Cell-Vessel Interaction Force

In our ABM, we assume that the centres of blood vessels do not move and that contact forces from neighbouring cells act on their vessel walls, causing deformation. For simplicity, we assume that the vessel is circular, and that its radius changes in response to the applied forces from neighbouring cells. We model the contact force between a cell and neighbouring vessel by assuming that a non-linear spring connects the cell's centre to the closest point on the vessel wall.

Suppose that cell i and vessel k are neighbours and have target equilibrium radii R_i and R_k respectively. They are connected by a non-linear spring, with natural spring length $L_{ik} = R_i + R_k$. In most cases $R_i = R_c$, unless the cell is growing (see Section S1.1.2) or dying (Section S1.1.3). By contrast, $R_k = R_k(t)$ evolves in a manner described below (see Section S1.3.3). The spring force $\vec{\mathbf{F}}_{ik}^{cv}$ between cell i and vessel k is given by:

$$|\vec{\mathbf{F}}_{ik}^{cv}| = \begin{cases} -2\mu R_k \ln\left(\frac{|\vec{\mathbf{r_i}} - \vec{\mathbf{r_k}}| - R_i}{R_k}\right) & \text{if } |\vec{\mathbf{r_i}} - \vec{\mathbf{r_k}}| < L_{ik}, \\ 0 & \text{otherwise,} \end{cases} \tag{10}$$

where μ is the spring stiffness parameter as in Equation (9). If the distance between the vessel and cell is less than the equilibrium length L_{ik}, then this force is repulsive and acts in the direction of the vector connecting the centres of cell i and vessel k; if the distance exceeds L_{ik} then the cell-vessel contact force is negligible. By Newton's Third Law, the force exerted on cell i by vessel k is equal and opposite to that applied by vessel k on cell i ($\vec{\mathbf{F}}^{cv}_{ik} = -\vec{\mathbf{F}}^{cv}_{ki}$).

S1.3.2 Tumour Cell Force Balance

Following existing models (e.g. [S4, S5]), the movement of cell centres is determined by solving a force balance equation. For a cell of mass m_i, whose centre is located at position $\vec{\mathbf{r}}_i$, the forces acting on it are: an applied force ($\vec{\mathbf{F}}^{\text{app}}_i$) due to mechanical interactions with neighbouring cells and vascular agents (see Section S1.3.1), friction ($\vec{\mathbf{F}}^{\text{fric}}_i$) representing cell-ECM adhesions and Stokes' drag ($\vec{\mathbf{F}}^{\text{drag}}_i = -\nu\dot{\vec{\mathbf{r}}}_{\mathbf{i}}$, where ν is the Stokes' drag coefficient) accounting for resistance due to relative movement of the cells compared to the extracellular fluid and ECM.

The applied force $\vec{\mathbf{F}}^{\text{app}}_i$ on cell i is the sum of contact forces due to interactions with neighbouring cells and vessels. It can be written as:

$$\vec{\mathbf{F}}^{\text{app}}_i = \sum_{\substack{j\in\{\mathcal{N}_i\}\\ j=\text{cell}}} \vec{\mathbf{F}}^{cc}_{ij} + \sum_{\substack{k\in\{\mathcal{N}_k\}\\ k=\text{vessel}}} \vec{\mathbf{F}}^{cv}_{ik} \tag{11}$$

where $\{\mathcal{N}_i\}$ is the set of agents within $3\hat{R}_c$ of cell i. $\vec{\mathbf{F}}^{cc}_{ij}$ and $\vec{\mathbf{F}}^{cv}_{ik}$ are the cell-cell and cell-vessel contact forces defined in Section S1.3.1.

We encode the effect of cell-ECM adhesion in $\vec{\mathbf{F}}^{\text{fric}}$. Cell-ECM attachments should anchor a cell when applied forces are small (below a limiting threshold value, $\mathcal{F}^{\text{friction}}$). In this regime $\vec{\mathbf{F}}^{\text{fric}}$ behaves as a static friction which precisely balances the applied forces to prevent cell movement. When applied forces exceed $\mathcal{F}^{\text{friction}}$ cell-ECM attachments break. In this case, the friction force is dynamic, with constant magnitude equal to $\mathcal{F}^{\text{friction}}$, and acts directly opposite to $\vec{\mathbf{F}}^{\text{app}}$. Dynamic friction accounts for the resistance to motion due to the continuous formation and breaking of bonds to the ECM.

Combining these effects, we assume that cell i experiences a friction force $\mathcal{F}^{\text{fric}}_i$ of the form:

$$\vec{\mathbf{F}}^{\text{fric}}_i = \begin{cases} -\vec{\mathbf{F}}^{\text{app}}_i & \text{if } |\vec{\mathbf{F}}^{\text{app}}_i| < \mathcal{F}^{\text{friction}} \\ -\frac{\vec{\mathbf{F}}^{\text{app}}_i}{|\vec{\mathbf{F}}^{\text{app}}_i|}\mathcal{F}^{\text{friction}} & \text{otherwise} \end{cases} \tag{12}$$

where ν and $\mathcal{F}^{\text{friction}}$ are a damping coefficient and friction strength parameters and describing a cell's resistance to motion due to interactions with the surrounding ECM.

Following Newton's second law, we can write the force balance as follows:

$$\underbrace{0 = m_i\ddot{\vec{\mathbf{r}}}_{\mathbf{i}}}_{\text{Viscous limit}} = \vec{\mathbf{F}}^{\text{app}}_i - \underbrace{\nu\dot{\vec{\mathbf{r}}}_{\mathbf{i}}}_{\text{Stokes' drag}} + \vec{\mathbf{F}}^{\text{fric}}_i \tag{13}$$

By taking the overdamped, viscous limit in which inertial effects can be neglected and rearranging Equation (13), we obtain the equation of motion for tumour cell i:

$$\nu\frac{d\vec{\mathbf{r}}_{\mathbf{i}}}{dt} = \vec{\mathbf{F}}^{\text{app}}_i + \vec{\mathbf{F}}^{\text{fric}}_i \tag{14}$$

S1.3.3 Blood Vessel Force Balance

In our model, vessels do not move; instead their outer wall deforms in response to the external pressure associated with contact forces from neighbouring cells. We estimate the external pressure P_i exerted on the outer wall of vessel i as follows. Let N_c be the number of cells neighbouring vessel i, and let $\vec{\mathbf{F}}^{cv}_{ji}$ be the force exerted by cell j on vessel i. We assume that the force exerted by each neighbouring cell is distributed uniformly around the vessel wall and, hence, that P_i can be written as:

$$P_i = \frac{1}{N_c} \sum_{j}^{N_c} \frac{1}{L_0} \vec{\mathbf{F}}^{cv}_{ji} \cdot \frac{\vec{\mathbf{r_i}} - \vec{\mathbf{r_j}}}{|\vec{\mathbf{r_i}} - \vec{\mathbf{r_j}}|} \tag{15}$$

where L_0 is the length of the cell-vessel interface (without loss of generality, we fix $L_0 = 1$ since cell boundaries are poorly defined in cell-centre models).

The difference between the external pressure P_i and internal pressure $P^\star$ results in a force on the vessel wall of magnitude $L_0 (P_i - P^\star)$ which acts to occlude the vessel if $P_i > P^\star$ and dilates the vessel if the external pressure is reduced ($P_i < P^\star$). Balancing this force with a damping force with damping coefficient ν_r and assuming that the vessel radius cannot exceed a maximum value of $\hat{R}_{\max}$, we can write the equation of motion for vessel radius $R_i = R_i(t)$:

$$\begin{aligned} \nu_r \frac{dR_i}{dt} &= -\, L_0 \left(P_i - P^\star\right) \mathcal{H} \left(\hat{R}_{\max} - R_i\right), \\ \text{where } \mathcal{H}(x) &= \begin{cases} 1 \text{ if } x > 0, \\ 0 \text{ otherwise.} \end{cases} \end{aligned} \tag{16}$$

S1.4 1D Off-Lattice System Demonstrating Impact of Force Laws

The 1D off-lattice system presented in Section 3.1 consists of a 1D chain of 100 cells, evenly spaced in the domain $x \in [0, 100]$, whose positions are determined by solving the associated equations of motion, as described above (see Section S1.3.2). We assume that the cells do not divide, and cannot become hypoxic or die. This enables us to focus only on mechanical interactions. To study the effect of the friction force, we slowly compress the cells by steadily moving the left hand boundary from $x = 0$ to $x = 20$, whilst holding the right hand boundary fixed at $x = 100$. The domain is compressed over a period of 80 hours, which is sufficiently slow that the cells remain at quasi-equilibrium with no oscillations forming. The left hand boundary is held fixed at $x = 20$ for a further 20 hours ($80 < t \leq 100$ hours). The final state of the system is analysed in Section 3.1.

S1.5 Default Values of Model Parameters

Table A: **Default Values of ABM Parameters** We do not compare our model to experimental data and, therefore, parameter values are chosen to generate behaviours which are in qualitative agreement with biological observations. The impact on the behaviour of the ABM of parameters marked with * is explored via parameter sweeps.

Symbol	Parameter	Parameter value	Dimensional range	Notes
dt	Timestep	1/120	1/120 (hours)	[S6]
D_ω	Oxygen Diffusion Coefficient	1	1750 (μm^{2}s^{-1})	Assumed
α	Oxygen Source Coefficient	5	- (mol vessel^{-1}s^{-1})	Assumed
κ	Oxygen Consumption Coefficient	0.03	- (mol cell^{-1}s^{-1})	Assumed*
λ_ω	Oxygen Decay Rate	0.03	- (mol s^{-1})	Assumed
$\overline{\tau}$	Average Tumour Cell Cycle Duration	24	24 (hours)	[S4]
τ_{apop}	Apoptosis Duration	48	48 (hours)	[S4]
η	Contact Inhibition Threshold	0	-	[S4, S5]*
ω_h	Tumour Hypoxic Concentration	0.2	- (mol)	[S4]*
ω_n	Tumour Necrotic Concentration	0.01	- (mol)	[S4, S7]
μ	Meineke SpringStiffness	5	$3-50$ (μg R_t^{-1} h^{-2})	[S6, S8]*
$\hat{R}_c$	Tumour Cell Target Radius	0.5	$7-12$ (μm)	[S4, S8]
δ	Intercellular Adhesion	5	-	[S4–S6]
ν	Damping Constant	1	0.4 (N m s^{-1})	[S6, S9]*
$\mathcal{F}^{\text{friction}}$	Friction Strength	2	- (N)	Assumed*
$P^\star$	Vessel Pressure	3	-	Assumed*
$\hat{R}_{\text{min}}$	Vessel Minimum Radius	0	-	Assumed
$\hat{R}_{\text{max}}$	Vessel Maximum Radius	0.75	-	Assumed
$\hat{R}_{\text{occ}}$	Vessel Occlusion Threshold	0.56	-	Assumed*
ν_r	Vessel Radius Damping	10	-	Assumed*
τ_{prune}	Vessel Pruning Duration	6	- (hours)	Assumed
ω_{angio}	Angiogenesis Oxygen Threshold	0.1	- (mol)	Assumed*
τ_{angio}	Angiogenesis Duration	12	- (hours)	Assumed
D_{angio}	Sprouting Distance	8	- (μm)	Assumed
P_{angio}	Angiogenesis Probability	0.2	-	Assumed*

References

[S1] Cooper, F. R. *et al.* Chaste: Cancer, Heart and Soft Tissue Environment. *Journal of open source software* **5,** 1848. doi:10.21105/joss.01848 (Mar. 2020).

[S2] Mirams, G. R. *et al.* Chaste: An Open Source C++ Library for Computational Physiology and Biology. en. *PLOS Computational Biology* **9.** Publisher: Public Library of Science, e1002970. doi:10.1371/journal.pcbi.1002970 (Mar. 2013).

[S3] Pitt-Francis, J. *et al.* Chaste: A test-driven approach to software development for biological modelling. *Computer Physics Communications. 40 YEARS OF CPC: A celebratory issue focused on quality software for high performance, grid and novel computing architectures* **180,** 2452–2471. doi:10.1016/j.cpc.2009.07.019 (Dec. 2009).

[S4] Bull, J. A., Mech, F., Quaiser, T., Waters, S. L. & Byrne, H. M. Mathematical modelling reveals cellular dynamics within tumour spheroids. en. *PLOS Computational Biology* **16.** Publisher: Public Library of Science, e1007961. doi:10.1371/journal.pcbi.1007961 (Aug. 2020).

[S5] Bull, J. A. & Byrne, H. M. Quantification of spatial and phenotypic heterogeneity in an agent-based model of tumour-macrophage interactions. en. *PLOS Computational Biology* **19,** e1010994. doi:10.1371/journal.pcbi.1010994 (Mar. 2023).

[S6] Osborne, J. M., Fletcher, A. G., Pitt-Francis, J. M., Maini, P. K. & Gavaghan, D. J. Comparing individual-based approaches to modelling the self-organization of multicellular tissues. en. *PLOS Computational Biology* **13.** Publisher: Public Library of Science, e1005387. doi:10.1371/journal.pcbi.1005387 (Feb. 2017).

[S7] Grimes, D. R., Kelly, C., Bloch, K. & Partridge, M. A method for estimating the oxygen consumption rate in multicellular tumour spheroids. *Journal of The Royal Society Interface* **11.** Publisher: Royal Society, 20131124. doi:10.1098/rsif.2013.1124 (Mar. 2014).

[S8] Laget, S. *et al.* Technical Insights into Highly Sensitive Isolation and Molecular Characterization of Fixed and Live Circulating Tumor Cells for Early Detection of Tumor Invasion. en. *PLOS ONE* **12.** Publisher: Public Library of Science, e0169427. doi:10.1371/journal.pone.0169427 (Jan. 2017).

[S9] Pathmanathan, P. *et al.* A computational study of discrete mechanical tissue models. en. *Physical Biology* **6,** 036001. doi:10.1088/1478-3975/6/3/036001 (Apr. 2009).
