# Supplementary material for: Exploring the relationship between vascular remodelling and tumour growth using agent-based modelling
Source: PLoS Comput Biol. 2026 May 15;22(5):e1012967. doi: 10.1371/journal.pcbi.1012967 (PMC13354106; doi:10.1371/journal.pcbi.1012967)
Supplement: S2 Text — (PDF) [file pcbi.1012967.s002.pdf]

S2 Parameter Sweeps

S2.1 Vascular Tumour Simulations

S2.1.1 Vessel Pressure and Friction Strength 2-Parameter Sweep

When we conduct our initial 2-parameter sweep, all parameters are held fixed, except $\mathcal{F}^{\text{friction}}$ and $P^{\star}$. Recalling that $\mathcal{F}^{\text{friction}}$ represents the maximum friction force a cell can experience, and $P^{\star}$ controls a vessel's sensitivity to external forces, this parameter sweep is designed to investigate the effect of including a friction force on vascular remodelling.

Table A: **Protocol for friction and vessel pressure parameter sweep.** Summary of parameter combinations explored in $P^{\star} - \mathcal{F}^{\text{friction}}$ parameter sweep. Every pair of values of $P^{\star}$ and $\mathcal{F}^{\text{friction}}$ was considered for every combination of configuration and random seeds.

Parameter	Values
Vessel Pressure ($P^{\star}$)	0.5, 1, 1.5, 2, 2.5, 3, 3.5, 4, 4.5, 5
Friction Strength ($\mathcal{F}^{\text{friction}}$)	0, 0.5, 1, 1.5, 2, 2.5, 3, 3.5, 4, 4.5, 5
Configuration Seed	*0, 1, 2, 3*
Random Seed	*0, 1, 2, 3*

The parameter ranges explored are summarised in Table A. We consider 10 values of $P^{\star}$ and 11 values of $\mathcal{F}^{\text{friction}}$, resulting in 110 combinations. All other model parameters are fixed at the default values stated in S1 Text. Each of the 110 parameter combinations is used to simulate tumours for 4 initial vessel configurations, and each of these combinations is repeated 4 times with different random seeds, giving a total of 1760 simulated tumours.

S2.1.2 Tumour Hypoxia Sensitivity and Vessel Pressure 2-Parameter Sweep

We perform a second 2-parameter sweep in order to investigate the impact of vessel remodelling on tumour growth. We varied P^{*}, the vessel pressure, and ω_h, the threshold oxygen concentration below which the cells become hypoxic and halt proliferation.

Table B: **Protocol for tumour hypoxia sensitivity and vessel pressure parameter sweep.** Details of parameter combinations explored in $P^{\star} - \omega_h$ parameter sweep. Every combination of values of $P^{\star}$ and ω_h is combined with each pair of configuration and random seeds.

Parameter	Values
Vessel Pressure ($P^{\star}$)	0.5, 1, 1.5, 2, 2.5, 3, 3.5, 4, 4.5, 5
Tumour Hypoxia Sensitivity (ω_h)	0.01, 0.1, 0.2, 0.3, 0.4, 0.5, 0.6
Configuration Seed	*0, 1, 2, 3*
Random Seed	*0, 1, 2, 3*

The parameter ranges we consider are summarised in Table B. Each combination of 10 values of $P^{\star}$ and 7 values of ω_h is used as a parameter set, giving a total of 70 combinations. As for the previous parameter sweep, all other parameters are fixed at the default values given in S1 Text. Each parameter set is used to simulate tumours with 4 different initial vessel configurations and 4 random seeds, giving a total of 1,120 simulations.

S2.1.3 Multidimensional Latin Hypercube Parameter Sweep

In order to efficiently explore a larger parameter space, we perform a multidimensional Latin hypercube sweep, in which eight model parameters are simultaneously varied. Latin hypercube sampling permits uniform sampling of a large region of parameter space and is used here to establish the robustness of trends identified in the 2-parameter sweeps when other parameters vary.

The parameters varied in our Latin hypercube are summarised in Table C. Each parameter is sampled from either a either uniform ($\mathcal{U}$) or a normal ($\mathcal{N}(\mu, \sigma)$) distribution. The distributions are truncated between stated intervals. We chose to sample from normal distributions when a parameter would usually be fixed in existing models [S1–S3], but we expect may have an effect on simulations.

Table C: **Protocol for Latin hypercube parameter sweep.** Each parameter is sampled from either a uniform ($\mathcal{U}$) or normal ($\mathcal{N}(\mu, \sigma)$) distribution with mean μ and standard deviation σ, and truncated to an interval of interest. 5000 parameter sets are drawn and repeated for each of the 4 configuration seeds.

Parameter	Distribution	Interval
Oxygen Consumption Coefficient (κ)	$\mathcal{N}(\mu = 0.03, \sigma = 0.05)$	$(0, 1]$
Tumour Hypoxia Sensitivity (ω_h)	$\mathcal{N}(\mu = 0.2, \sigma = 0.2)$	$(0, 0.8]$
Vessel Pressure ($P^\star$)	$\mathcal{U}$	$[1, 5]$
Friction Strength ($\mathcal{F}^{\text{friction}}$)	$\mathcal{U}$	$[0, 5]$
Tumour Quiescence Volume (η)	$\mathcal{N}(\mu = 0, \sigma = 0.2)$	$[0, 0.8]$
Vessel Occlusion Threshold (R_{occ})	$\mathcal{U}$	$[0.375, 0.75)$
Damping Constant (ν)	$\mathcal{N}(\mu = 1, \sigma = 4)$	$[0, 10]$
Spring Stiffness (μ)	$\mathcal{N}(\mu = 5, \sigma = 4)$	$[0, 10]$
Configuration Seed	*0, 1, 2, 3*	
Random Seed	*0*	

5000 parameter sets are sampled from the 8-dimensional Latin hypercube, varying κ, ω_h, $P^\star$, $\mathcal{F}^{\text{friction}}$, η, R_{occ}, ν and μ. Parameters are sampled from either a uniform distribution ($\mathcal{U}$), or a truncated normal distribution $\mathcal{N}$ in order to focus sampling around a mean value (see Table C). For each parameter set simulations are repeated with 4 initial vessel configurations, giving a total of 20,000 simulations.

S2.2 Vessel Pressure and Angiogenesis 2-Parameter Sweep with Radiotherapy

To study the effect of radiotherapy, 4 tumours are seeded in a vascularised domain at $t = 0$ and allowed to grow for 21 days. They are then treated with a single, 6 Gray dose of radiotherapy. The simulations are continued for a further 21 days to observe the tumour's post-radiotherapy growth dynamics.

Table D: **Protocol for vessel pressure and angiogenesis parameter sweep with radiotherapy.** Summary of parameter combinations used to perform the $P^\star - \omega_{\text{angio}}$ radiotherapy sweep. Every combination of $P^\star$ and ω_{angio} is combined with each pair of configuration and random seed.

Parameter	Values
Vessel Pressure ($P^\star$)	0.5, 1.0, 1.5, 2.0, 2.5, 3.0, 3.5, 4.0, 4.5, 5.0
Angiogenesis Oxygen Threshold (ω_{angio})	0.05, 0.1, 0.15, 0.2, 0.25, 0.3, 0.35, 0.4, 0.45, 0.5
Configuration Seed	*0, 1, 2, 3*
Random Seed	*0, 1, 2, 3*

The parameter ranges explored are summarised in Table D. Every combination of values of $P^\star$ and ω_{angio} is used, giving 100 parameter sets (with all other parameters fixed at the default values specified in Table A in S1 Text). Each parameter set is used to simulate tumours repeated with 4 initial configuration seeds and 4 random seeds, giving a total of 1600 simulations.

References

[S1] Bull, J. A., Mech, F., Quaiser, T., Waters, S. L. & Byrne, H. M. Mathematical modelling reveals cellular dynamics within tumour spheroids. en. *PLOS Computational Biology* **16.** Publisher: Public Library of Science, e1007961. doi:`10.1371/journal.pcbi.1007961` (Aug. 2020).

[S2] Bull, J. A. & Byrne, H. M. Quantification of spatial and phenotypic heterogeneity in an agent-based model of tumour-macrophage interactions. en. *PLOS Computational Biology* **19,** e1010994. doi:`10.1371/journal.pcbi.1010994` (Mar. 2023).

[S3] Osborne, J. M., Fletcher, A. G., Pitt-Francis, J. M., Maini, P. K. & Gavaghan, D. J. Comparing individual-based approaches to modelling the self-organization of multicellular tissues. en. *PLOS Computational Biology* **13.** Publisher: Public Library of Science, e1005387. doi:`10.1371/journal.pcbi.1005387` (Feb. 2017).
