# Supplementary material for: Exploring the relationship between vascular remodelling and tumour growth using agent-based modelling
Source: PLoS Comput Biol. 2026 May 15;22(5):e1012967. doi: 10.1371/journal.pcbi.1012967 (PMC13354106; doi:10.1371/journal.pcbi.1012967)
Supplement: S3 Text — (PDF) [file pcbi.1012967.s003.pdf]

S3 Additional Model Analysis

S3.1 Basic Tumour Morphology Metrics

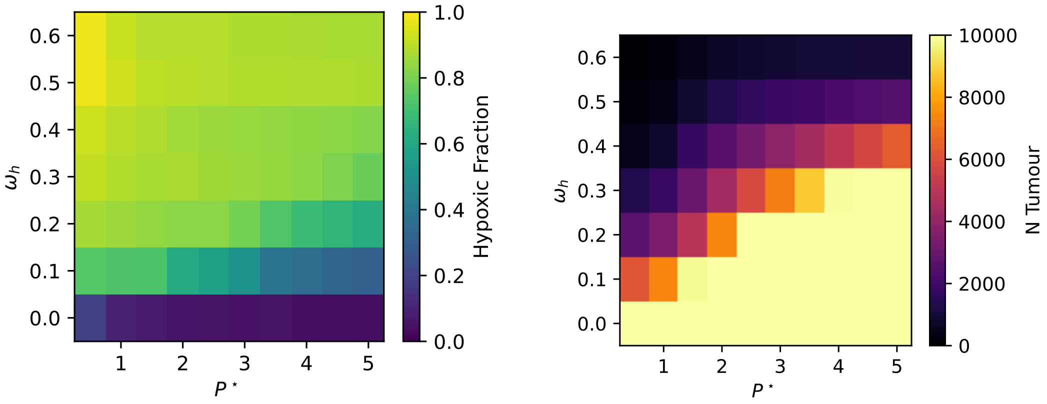

Figure A: **Variation of tumour hypoxic fraction and tumour size for the two-parameter sweep in which $P^\star$ and ω_h vary** (Section S2.1.2 in S2 Text). a) Heatmap showing how the hypoxic proportion of the tumour at the end of each simulation changes as P^* and ω_h vary (averaged over 16 repetitions). The hypoxic fraction is positively correlated with ω_h (hypoxic sensitivity) and negatively correlated with $P^\star$ (determinant of oxygen supply). b) Heatmap showing how the average tumour mass at the end of each simulation changes as P^* and ω_h vary (averaged over 16 repetitions). Comparing a) and b), we note the largest tumours have the smallest hypoxic fraction and are generated in parameter regimes with low values of ω_h.

In Figure A we show how two additional tumour metrics (the hypoxic fraction and the number of tumour cells at the end of each simulation) change as $P^\star$ and ω_h vary.

S3.2 Radiotherapy Susceptibility across 2-Parameter Sweep

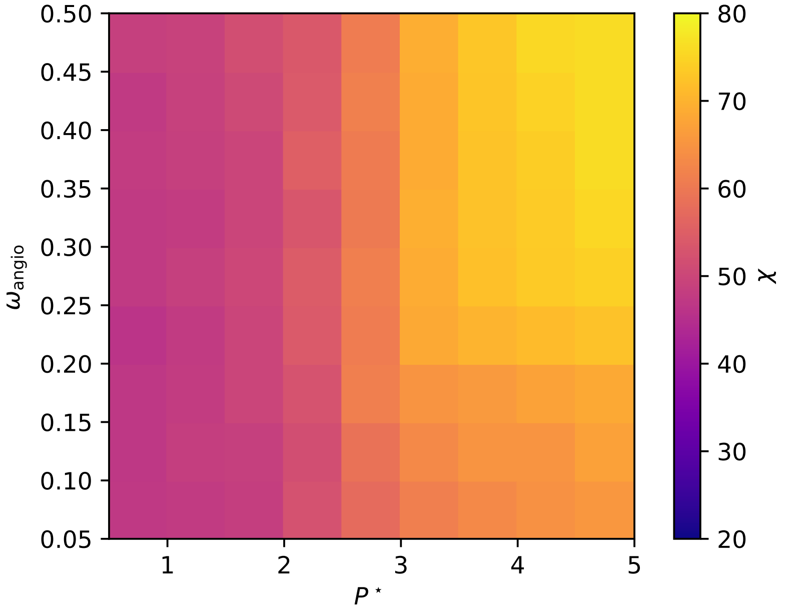

Figure B: **Effect of vessel parameters on radiotherapy susceptibility** Percentage of tumour killed (χ) in single dose of radiotherapy across $P^\star\omega_{\text{angio}}$ parameter sweep (Section S2.2 in S2 Text). This figure accompanies Fig 6B showing χ across the 2-parameter sweep. We see that the variation in χ is due to $P^\star$, and ω_{angio} has comparatively no effect on χ.

Figure B accompanies Fig 6B, and shows the change in χ after radiotherapy as $P^\star$ and ω_{angio} vary in a parameter sweep.
