# Supplementary material for: Exploring the relationship between vascular remodelling and tumour growth using agent-based modelling
Source: PLoS Comput Biol. 2026 May 15;22(5):e1012967. doi: 10.1371/journal.pcbi.1012967 (PMC13354106; doi:10.1371/journal.pcbi.1012967)
Supplement: Attachment — Submitted filename: Response to Reviewers.pdf [file pcbi.1012967.s006.pdf]

Reference: Manuscript ID PCOMPBIOL-D-25-00500

Exploring the relationship between vascular remodelling and tumour growth using agent-based modelling

Nicholas Fan, Joshua A. Bull and Helen M. Byrne

Response to Reviewers

We thank the reviewers for their helpful comments. We have revised the manuscript in line with their suggestions, and include below a point-by-point response to the comments that they raised, together with associated changes to the text. We hope that the revised paper addresses their comments and is now suitable for publication in *Plos Computational Biology*.

Response to Reviewer 1

Comment 1. Especially in the introduction, it would be important to make it clear that "pressure" refers to solid pressure, or solid stress, and that fluid pressure is not considered in the model.

Response: We have revised the text to clarify that 'pressure' refers to the 'solid pressure' associated with cell-cell or cell-vessel interactions and that fluid pressure is neglected in the current model. We note also that including fluid pressure in the model represents an interesting direction for future investigation.

Manuscript changes:

- Page 2: "...mechanical stress generated by rapid tumour growth increases the solid pressure that the tumour cells exert on blood vessels..."
- Page 2: "...friction is necessary for the generation of solid pressure gradients that are sufficiently large to drive vessel occlusion: when Stokes' drag dominates, solid pressure gradients dissipate across the tissue and vessel compression is negligible. By contrast, as the strength of the friction force increases, larger solid pressure gradients form and drive vessel occlusion. While our model provides a mechanism to study the impact of solid pressure on vessel occlusion, we note that the effects of fluid pressure within the extracellular fluid are neglected explicitly."
- Page 7: "We assume that vessel deformation is driven by the difference between the external solid ~~applied~~ pressure P_i, and the internal vessel pressure $P^{\star}$."
- Page 7: "...we further assume that the mean force per unit length exerted by cells surrounding a blood vessel approximates the solid ~~external~~ pressure P_i experienced by the vessel."
- Page 20: "...In future work it would be interesting to consider the impact that the extracellular fluid exerts on the mechanical pressure in the tumour [1–3]. When tumour cells are killed they disintegrate into extracellular fluid and residual cellular fragments are cleared by phagocytic immune cells, including macrophages. This process can generate

high interstitial fluid pressure in the tumour core, which drives movement of extracellular fluid out of the tumour [4, 5]."

Comment 2. The authors should clarify what happens at the tumor boundary. Do the cells there experience less friction, or is the constraint imposed by surrounding tissue being represented somehow?

Response:

We are unsure whether the comment refers to the boundary of the computational domain or the boundary that defines the spatial extent of the tumour cells.

In regard to the simulation boundary: in Methods we clarify that simulations are halted before reaching the edge of the computational domain.

In regard to the tumour boundary: As with many existing ABMs [6–8], we model the mechanical effect of the surrounding tissue and extracellular matrix implicitly. Existing ABMs implement a Stokes' drag force that acts on the cells to account for such mechanical interactions. Here, we introduce an additional friction force whose magnitude and direction depend on the forces applied to a cell by its neighbours (see Section 2.1.2). As part of the circularity analysis (see Section 2.3.2) we approximate the tumour boundary using alpha shapes, however this does not influence the behaviour of individual cells which depends only on their local environment. In future work, it would be interesting to explicitly model the surrounding tissue and extracellular matrix and the forces that they exert on cells at the tumour boundary; such extensions are, however, beyond the scope of this paper. We have extended our discussion to include this potential direction for future work.

Manuscript changes:

- Page 7: Equation 5 is solved at discrete timesteps using a forward Euler approach, noting that all simulations are halted before cells reach the boundary of the computational domain.
- Page 19: "Our simulations show that including this friction force, together with Stokes' drag, prevents rapid pressure dissipation, and enables vessel occlusion due to mechanical pressure. We note that, as described, this force does not account for resistive forces at the boundary of a growing tumour from the surrounding tissue; we do not explicitly model these effects. Further studies are needed to explore whether constraints imposed by the surrounding tissue could be accounted for by varying the friction force applied to cells close to the tumour boundary (which may be approximated using alpha shapes, see Section 2.3.2)."

Comment 3. An outstanding question in the field relates to how solid stresses are affected by cell invasion (see, e.g. https://doi.org/10.1038/s41551-018-0334-7). Can the model be modified to include active cell migration to address this question?

Response: We agree that cell invasion is an important process that is regulated/influenced by solid stresses in the surrounding ECM. Our model focuses on solid pressure inside the tumour driving vessel occlusion. In particular, our model does not track solid pressure in the surrounding tissue. The impact of the ECM on tumour invasion is modelled indirectly via the friction force acting on individual tumour cells. Significant model extensions would be needed to study how invasion is affected by solid stress in the surrounding ECM.

In the original manuscript we explain how our model could be extended to account for ECM explicitly. We note that this would enable study of the solid stress patterns that arise from different forms of cell migration (e.g. following epithelial mesenchymal transition). We have extended our discussion to clarify the above points.

Manuscript changes:

- Page 20: ~~Given the importance of cell-ECM interactions in generating mechanical stress within solid tumours [9, 10], and motivated by existing models of cell-ECM interactions [11, 12] (reviewed in [13, 14]), it would also be interesting in future work to incorporate the ECM into our model, to explicitly model the formation and breaking of cell-ECM bonds of adhesion and study their impact on cell movement and ECM deformation.~~ In the future, it would also be interesting to incorporate a model of extracellular matrix (ECM) into our work [11–16], to explicitly capture the formation and breaking of cell–ECM adhesion bonds and to study their effects on cell migration and ECM deformation [9, 10]. Active cell migration plays a key role in cancer invasion and is known to be influenced by solid stress [17]. Extending our model to investigate active cell migration into the surrounding ECM and stroma would therefore be of great interest. However, additional work is needed to better understand the underlying physics of cell motility, particularly the role of epithelial–mesenchymal transition in this process [18, 19].

Comment 4. The inclusion of both the drag force and the friction force is confusing. Once the cell is moving, how do these differ, and are they both necessary? Also, Figure 1b could be more detailed to clarify how the friction force is working in the different regimes (stuck vs. moving).

Response: We agree that the distinction between the friction force when a cell is moving and when it is static is difficult to explain. We have revised the text and Figure 1b to more clearly explain and motivate this distinction.

Manuscript changes:

- Page 7: "If $|\vec{\mathbf{F}}^{\text{app}}| < \mathcal{F}^{\text{friction}}$ then the applied force is exactly balanced by friction ($\vec{\mathbf{F}}^{\text{fric}} = -\vec{\mathbf{F}}^{\text{app}}$) and the cell does not move. In this case, the friction force corresponds to a static friction force and the parameter $\mathcal{F}^{\text{friction}}$ represents the magnitude of the limiting friction force. If $|\vec{\mathbf{F}}^{\text{app}}| \geq \mathcal{F}^{\text{friction}}$ then the limiting friction force is unable to balance the applied force and the cell moves. In this case, we assume that two effects contribute to the net resistive force: continual formation and breaking of cell-ECM attachments, and resistance to movement through viscous extracellular fluid. We assume that the resistance force associated with cell-ECM attachments has the same magnitude as limiting friction $\mathcal{F}^{\text{friction}}$, and acts in the opposite direction to the applied force ($\left(\vec{\mathbf{F}}^{\text{fric}} = -\frac{\vec{\mathbf{F}}^{\text{app}}}{|\vec{\mathbf{F}}^{\text{app}}|}\mathcal{F}^{\text{friction}}\right)$). The resistance force associated with cell movement through the extracellular fluid is modelled as a Stokes' drag force; similar drag terms have been used in other ABMs [6, 20–23]. ~~then the friction force becomes dynamic, with fixed magnitude $\mathcal{F}^{\text{friction}}$, acting opposite to $\vec{\mathbf{F}}^{\text{app}}$. Biologically, the dynamic friction force is the resistance to motion due to continual formation and breaking of cell-ECM attachments. Here we assume that cell-ECM attachments break and form on timescales which are much faster the timescale of cell movement.~~"

Comment 5. Such a model would be straightforward to extend to 3D. The authors should comment on the potential limitations of the 2D simulations.

Response: We agree that some aspects of the extension of our model from 2D to 3D would be straightforward (eg tumour cells and the distribution of oxygen). However other aspects would require substantial model development. In particular, extending our description of blood vessels as circular cross sections (similar to [24]) to a 3D network of interconnected blood vessels would require substantial change. The vasculature could be represented as a complex geometry [8] or as a network of connected cylinders [7], but significant development would still be needed to account for vessel remodelling and, in particular, to account for occlusion due to solid stress. Alternatively, we could extend Microvessel CHASTE to 3D [25], but, again, significant work would be needed to account for solid stress driven vessel occlusion. Utilising Microvessel CHASTE would, however, enable us to model blood flow through the dynamically remodelled vascular network.

We have extended our discussion to clarify the above points.

Manuscript changes:

- Page 20: "While our use of the Chaste computational framework would facilitate the extension of the tumour growth and oxygen diffusion aspects of our model to 3D, representing the vasculature in 3D presents considerable computational challenges. [7] and [8] offer two approaches for representing 3D vasculature, however both would require significant extensions to account for vessel occlusion and pruning."

- Page 20: "Future work might relax our simplified view of the vasculature as a series of point sources of oxygen, instead modelling a dynamic network of connected blood vessels [26–33]. Microvessel CHASTE [25], a CHASTE module for studying blood flow through vascular networks, could be incorporated to more accurately capture the interplay between tumour growth and local haematocrit levels. It would be interesting to use this to study 'collateral' effects, in which occlusion of upstream vessels impacts downstream vessels. ~~We could relax our simplified view of the vasculature as a series of point sources of oxygen. Following [25, 26, 30, 34, 35], we could instead represent it as a dynamic network of connected vessels and model blood flow and oxygen transport through the network.~~"

Response to Reviewer 2

Comment 1. Methods: Regarding the model's approximation of "Dynamic Oxygen Supply", while varying vessel diameters are accounted for, how are the authors accounting for the fact that arterioles, capillaries, and venules contain blood whose oxygen tensions (i.e. P02) differ?

Response: Our model assumes that the rate at which oxygen diffuses from each vessel is proportional to its radius, as a simple way of relating oxygen supply to vessel size. Developing more accurate models of the haematocrit distribution in vascular networks is an active and computationally challenging area of study. Providing a full description of this would require coupling our tumour growth model with a 3D model of vascular remodelling (including angiogenesis and vessel regression), alongside a model of haematocrit distribution within this evolving network. This would be an extremely exciting model, but is unfortunately beyond the scope of the current work. We have included additional text in the introduction and discussion sections to acknowledge the importance of varying oxygen tension throughout the vascular network.

Manuscript changes:

- Page 3: "...We assume further that changes in vessel cross-sectional area modulate both the supply of oxygen to the tissue and the rate at which the vessels are pruned from the system, a simplifying approximation which reflects oxygen tension varying between vessels of different sizes."
- Page 20: "Future work might relax our simplified view of the vasculature as a series of point sources of oxygen, instead modelling a dynamic network of connected blood vessels [26–33]. Microvessel CHASTE [25], a CHASTE module for studying blood flow through vascular networks, could be incorporated to more accurately capture the interplay between tumour growth and local haematocrit levels. This would enable us to study 'collateral' effects, in which occlusion of upstream vessels impacts downstream vessels. ~~We could also relax our simplified view of the vasculature as a series of point sources of oxygen. Following [25, 26, 30, 34, 35], we could instead represent it as a dynamic network of connected vessels and model blood flow and oxygen transport through the network.~~"

Comment 2. Methods: For the "Occlusion, Pruning and Angiogenesis", the authors are not accounting for the connectivity of the vasculature, such that if one vessels is occluded or pruned, it is likely that nearby "collateral" blood vessels (that are connected to the same upstream parent vessel as the occluded vessel) would experience increased blood flow, and they could dynamically alter their diameter, as a result. In the Discussion, the authors briefly mention this model limitation as a potential opportunity for future model extension, but it would be helpful to also state this important model assumption in the description of the model in the Methods section when it is first described.

Response: We agree, and now clearly state this simplifying assumption in the Methods section. We also explain how extending the model to represent the vasculature as a dynamic network of interconnected blood vessels, and simulating blood flow through the network, would enable us to study 'collateral' effects caused by vessel remodelling, although this would require substantial work beyond the scope of this paper.

Manuscript changes:

- Page 6: "We note that in practice, when a vessel is pruned, the surrounding vessels are likely to experience increased blood flow; for simplicity, this effect is neglected in the current model."
- Page 20: "Future work might relax our simplified view of the vasculature as a series of point sources of oxygen, instead modelling a dynamic network of connected blood vessels [26–33]. Microvessel CHASTE [25], a CHASTE module for studying blood flow through vascular networks could be incorporated to more accurately capture the interplay between tumour growth and local haematocrit levels. This would enable us to study 'collateral' effects, in which occlusion of upstream vessels impacts downstream vessels. ~~We could relax our simplified view of the vasculature as a series of point sources of oxygen. Following [25, 26, 30, 34, 35], we could instead represent it as a dynamic network of connected vessels and model blood flow and oxygen transport through the network.~~"

Comment 3. Methods: It is not clear why each simulation starts with 600 pruned vessels. One would assume that if there are only 4 tumor cells present initially, most if not all of the vessels would be "healthy".

Response: We apologise for this confusion. We have clarified that 'pruned' vessels are used to represent locations at which new vessels may form via angiogenesis.

Manuscript changes:

- Page 9: "Each simulation starts with 4 tumour cells seeded at the centre of a 1mm by 1mm square domain with 400 'healthy' blood vessels placed randomly without overlap. We initialise 600 additional locations at which new vessels can grow via angiogenesis; we term them 'Pruned' vessels (see Section 2.1.1)~~.and 600 'pruned' vessels are randomly placed in the domain without overlap, representing a well vascularised tissue. (see Section 2.1.1).~~"

Comment 4. Results: in 3.3 the authors explore how ω_h, the threshold oxygen concentration below which cells halt proliferation and become quiescent), affects tumor size, composition and morphology. With lower ω_h values, cells can withstand lower oxygen concentrations and remain viable at greater distances from blood vessels, leading to a compact tumor mass. Whereas with higher ω_h values, tumor cells are restricted to grow around the perfused blood vessels. They quantify the resulting tumor shape using a roundness score. It would seem that this prediction might be one that has (or could be) quantified in experimental or patient-derived pathology. Validation of this prediction by comparison to existing, publicly available data would strengthen the impact of this discovery.

Response: We agree that this would be a great way to test the practical utility of the roundness score and have extended our discussion to explain how spatial analysis could be used to validate our model against experimental data in future work. However spatial analysis of experimental data is a large and ongoing field [36–40] and further development of these methods, beyond the scope of this paper, is necessary before they can be used to validate our model.

Ideally this work would entail computing tumour roundness in longitudinal patient data, however such data tends to be from well developed tumours where mechanisms such as EMT,

ECM remodelling and immune infiltration are important [41]. Our model is instead representative of the early stages of tumour development, where tumour cell counts are low and vessel occlusion plays a key role in cancer development [42]. An alternative approach may be to investigate correlation between tumour roundness and response to radiotherapy, however this also requires substantial research beyond the scope of this paper.

We have extended our discussion with the above points.

Manuscript changes:

- Page 20: "In future work we could also compare our results with experimental data. Multiplex imaging methods [40] can locate and phenotype individual cell, paving the way for data that matches the spatial resolution of our model. The methods we have used to analyse our simulated data could be applied to such imaging data [36, 37, 43], and used for model parametrisation. Further development of such methods may enable validation of our model against experimental data, however suitable patient data is difficult to generate because our model is representative of small tumours at early stages of development whilst patient derived tumours are typically larger and well developed. Mechanisms neglected in the current model, such as immune infiltration, ECM remodelling and epithelial mesenchymal transition of cancer cells likely contribute to tumour morphology in such late stage tumours.

References

1. Tan, M., Song, B., Zhao, X. & Du, J. The role and mechanism of compressive stress in tumor. *Frontiers in Oncology* **14,** 1459313. doi:10.3389/fonc.2024.1459313 (2024).
2. Jain, R. K. Determinants of Tumor Blood Flow: A Review1. *Cancer Research* **48,** 2641–2658 (1988).
3. Jain, R. K. Vascular and interstitial barriers to delivery of therapeutic agents in tumors. *Cancer and Metastasis Reviews* **9,** 253–266. doi:10.1007/BF00046364 (1990).
4. Delarue, M. *et al.* Compressive Stress Inhibits Proliferation in Tumor Spheroids through a Volume Limitation. *Biophysical Journal* **107,** 1821–1828. doi:10.1016/j.bpj.2014.08.031 (2014).
5. Wu, M. *et al.* The effect of interstitial pressure on tumor growth: Coupling with the blood and lymphatic vascular systems. *Journal of Theoretical Biology* **320,** 131–151. doi:10.1016/j.jtbi.2012.11.031 (2013).
6. Bull, J. A. & Byrne, H. M. Quantification of spatial and phenotypic heterogeneity in an agent-based model of tumour-macrophage interactions. *PLOS Computational Biology* **19,** e1010994. doi:10.1371/journal.pcbi.1010994 (2023).
7. Duswald, T., Lima, E. A., Oden, J. T. & Wohlmuth, B. Bridging scales: A hybrid model to simulate vascular tumor growth and treatment response. *Computer Methods in Applied Mechanics and Engineering* **418,** 116566. doi:10.1016/j.cma.2023.116566 (2024).
8. Macnamara, C. K., Caiazzo, A., Ramis-Conde, I. & Chaplain, M. A. J. Computational modelling and simulation of cancer growth and migration within a 3D heterogeneous tissue: The effects of fibre and vascular structure. *Journal of Computational Science* **40,** 101067. doi:10.1016/j.jocs.2019.101067 (2020).
9. Deng, B. *et al.* Biological role of matrix stiffness in tumor growth and treatment. *Journal of Translational Medicine* **20,** 540. doi:10.1186/s12967-022-03768-y (2022).

10. Guillaume, L. *et al.* Characterization of the physical properties of tumor-derived spheroids reveals critical insights for pre-clinical studies. *Scientific Reports* **9.** Publisher: Nature Publishing Group, 6597. doi:10.1038/s41598-019-43090-0 (2019).

11. Chassonnery, P. *et al.* Fibre crosslinking drives the emergence of order in a three-dimensional dynamical network model. *Royal Society Open Science* **11,** 231456. doi:10.1098/rsos.231456 (2024).

12. Arellano-Tintó, J., Stepanova, D., Byrne, H. M., Maini, P. K. & Alarcón, T. *Multiscale modelling shows how cell-ECM interactions impact ECM fibre alignment and cell detachment* 2024. doi:10.1101/2024.12.05.627121.

13. Crossley, R. M. *et al.* Modeling the extracellular matrix in cell migration and morphogenesis: a guide for the curious biologist. *Frontiers in Cell and Developmental Biology* **12.** Publisher: Frontiers. doi:10.3389/fcell.2024.1354132 (2024).

14. Noël, V. *et al.* PhysiMeSS - a new physiCell addon for extracellular matrix modelling. *Gigabyte* **2024.** Publisher: GigaScience Press, gigabyte136. doi:10.46471/gigabyte.136 (2024).

15. Reinhardt, J. W. & Gooch, K. J. An Agent-Based Discrete Collagen Fiber Network Model of Dynamic Traction Force-Induced Remodeling. *Journal of Biomechanical Engineering* **140.** doi:10.1115/1.4037947 (2018).

16. Zheng, Y. *et al.* Modeling cell migration regulated by cell extracellular-matrix micromechanical coupling. *Physical Review E* **100,** 043303. doi:10.1103/PhysRevE.100.043303 (2019).

17. Seano, G. *et al.* Solid stress in brain tumours causes neuronal loss and neurological dysfunction and can be reversed by lithium. *Nature Biomedical Engineering* **3,** 230–245. doi:10.1038/s41551-018-0334-7 (2019).

18. Fortunato, I. C. *et al.* Single-cell migration along and against confined haptotactic gradients. *Nature Physics,* 1–10. doi:10.1038/s41567-025-03015-3 (2025).

19. Krummel, M. F., Friedman, R. S. & Jacobelli, J. Modes and Mechanisms of T cell Motility: Roles for Confinement and Myosin-IIA. *Current opinion in cell biology* **0,** 9–16. doi:10.1016/j.ceb.2014.05.003 (2014).

20. Bull, J. A., Mech, F., Quaiser, T., Waters, S. L. & Byrne, H. M. Mathematical modelling reveals cellular dynamics within tumour spheroids. *PLOS Computational Biology* **16.** Publisher: Public Library of Science, e1007961. doi:10.1371/journal.pcbi.1007961 (2020).

21. D'Antonio, G., Macklin, P. & Preziosi, L. An agent-based model for elasto-plastic mechanical interactions between cells, basement membrane and extracellular matrix. *Mathematical biosciences and engineering: MBE* **10,** 75–101. doi:10.3934/mbe.2013.10.75 (2013).

22. Ghaffarizadeh, A., Heiland, R., Friedman, S. H., Mumenthaler, S. M. & Macklin, P. PhysiCell: An open source physics-based cell simulator for 3-D multicellular systems. *PLOS Computational Biology* **14.** Publisher: Public Library of Science, e1005991. doi:10.1371/journal.pcbi.1005991 (2018).

23. Rocha, H. L. *et al.* A hybrid three-scale model of tumor growth. *Mathematical Models and Methods in Applied Sciences* **28.** Publisher: World Scientific Publishing Co., 61–93. doi:10.1142/S0218202518500021 (2018).

24. Scott, J. G., Fletcher, A. G., Anderson, A. R. A. & Maini, P. K. Spatial Metrics of Tumour Vascular Organisation Predict Radiation Efficacy in a Computational Model. *PLOS Computational Biology* **12.** Publisher: Public Library of Science, e1004712. doi:10.1371/journal.pcbi.1004712 (2016).

25. Grogan, J. A. *et al.* Microvessel Chaste: An Open Library for Spatial Modeling of Vascularized Tissues. *Biophysical Journal* **112.** Publisher: Elsevier, 1767–1772. doi:10.1016/j.bpj.2017.03.036 (2017).

26. Alarcón, T., Byrne, H. M. & Maini, P. K. A Multiple Scale Model for Tumor Growth. *Multiscale Modeling & Simulation* **3.** Publisher: Society for Industrial and Applied Mathematics, 440–475. doi:10.1137/040603760 (2005).

27. Owen, M. R., Alarcón, T., Maini, P. K. & Byrne, H. M. Angiogenesis and vascular remodelling in normal and cancerous tissues. *Journal of Mathematical Biology* **58,** 689–721. doi:10.1007/s00285-008-0213-z (2009).

28. Perfahl, H. *et al.* Multiscale Modelling of Vascular Tumour Growth in 3D: The Roles of Domain Size and Boundary Conditions. *PLOS ONE* **6.** Publisher: Public Library of Science, e14790. doi:10.1371/journal.pone.0014790 (2011).

29. McDougall, S. R., Anderson, A. R. A. & Chaplain, M. A. J. Mathematical modelling of dynamic adaptive tumour-induced angiogenesis: Clinical implications and therapeutic targeting strategies. *Journal of Theoretical Biology* **241,** 564–589. doi:10.1016/j.jtbi.2005.12.022 (2006).

30. Stéphanou, A., McDougall, S. R., Anderson, A. R. A. & Chaplain, M. A. J. Mathematical modelling of the influence of blood rheological properties upon adaptative tumour-induced angiogenesis. *Mathematical and Computer Modelling. Advances in Business Modeling and Decision Technologies [pp. 1-95]* **44,** 96–123. doi:10.1016/j.mcm.2004.07.021 (2006).

31. Bartha, K. & Rieger, H. Vascular network remodeling via vessel cooption, regression and growth in tumors. *Journal of Theoretical Biology* **241,** 903–918. doi:10.1016/j.jtbi.2006.01.022 (2006).

32. Lee, Y., Kouvroukoglou, S., McIntire, L. V. & Zygourakis, K. A cellular automaton model for the proliferation of migrating contact-inhibited cells. *Biophysical Journal* **69.** Publisher: Elsevier, 1284–1298. doi:10.1016/S0006-3495(95)79996-9 (1995).

33. Welter, M., Bartha, K. & Rieger, H. Emergent vascular network inhomogeneities and resulting blood flow patterns in a growing tumor. *Journal of Theoretical Biology* **250,** 257–280. doi:10.1016/j.jtbi.2007.09.031 (2008).

34. Alarcón, T., Byrne, H. M. & Maini, P. K. A cellular automaton model for tumour growth in inhomogeneous environment. *Journal of Theoretical Biology* **225,** 257–274. doi:10.1016/S0022-5193(03)00244-3 (2003).

35. Grogan, J. A., Maini, P. K., Pitt-Francis, J. & Byrne, H. M. *Simulating tumour vasculature at multiple scales* in *Proceedings of the 2014 6th International Advanced Research Workshop on In Silico Oncology and Cancer Investigation - The CHIC Project Workshop (IARWISOCI)* (2014), 1–4. doi:10.1109/IARWISOCI.2014.7034633.

36. Bull, J. A., Mulholland, E. J., Leedham, S. J. & Byrne, H. M. Extended correlation functions for spatial analysis of multiplex imaging data. *Biological Imaging* **4,** e2. doi:10.1017/S2633903X24000011 (2024).

37. Bull, J. A., Moore, J. W., Mulholland, E. J., Leedham, S. J. & Byrne, H. M. *MuSpAn: A Toolbox for Multiscale Spatial Analysis* Pages: 2024.12.06.627195 Section: New Results. 2024. doi:10.1101/2024.12.06.627195.

38. Parra, E. R. Methods to Determine and Analyze the Cellular Spatial Distribution Extracted From Multiplex Immunofluorescence Data to Understand the Tumor Microenvironment. *Frontiers in Molecular Biosciences* **8.** doi:10.3389/fmolb.2021.668340 (2021).

39. Andreou, C., Weissleder, R. & Kircher, M. F. Multiplexed imaging in oncology. *Nature Biomedical Engineering* **6,** 527–540. doi:10.1038/s41551-022-00891-5 (2022).

40. Lewis, S. M. *et al.* Spatial omics and multiplexed imaging to explore cancer biology. *Nature Methods* **18.** Publisher: Nature Publishing Group, 997–1012. doi:10.1038/s41592-021-01203-6 (2021).

41. Anderson, N. M. & Simon, M. C. The tumor microenvironment. *Current biology: CB* **30,** R921–R925. doi:10.1016/j.cub.2020.06.081 (2020).

42. Bi, S. *et al.* Three-dimensional characteristics of T cells and vasculature in the development of mouse esophageal cancer. *iScience* **27,** 111380. doi:10.1016/j.isci.2024.111380 (2024).

43. Bull, J. A. *et al.* *Integrating diverse statistical methods to analyse stage-discriminatory cell interactions in colorectal neoplasia* Pages: 2024.06.02.597010 Section: New Results. 2024. doi:10.1101/2024.06.02.597010.
