# Supplementary material for: Exploring the relationship between vascular remodelling and tumour growth using agent-based modelling
Source: PLoS Comput Biol. 2026 May 15;22(5):e1012967. doi: 10.1371/journal.pcbi.1012967 (PMC13354106; doi:10.1371/journal.pcbi.1012967)
Supplement: Peer review history

# Peer review: Decision Letter 0

**DOI:** 10.1371/journal.pcbi.1012967.r001

**Authors:** Feilim Mac Gabhann

21 Aug 2025

PCOMPBIOL-D-25-00500

Exploring the relationship between vascular remodelling and tumour growth using agent-based modelling

PLOS Computational Biology

Dear Dr. Fan,

Thank you for submitting your manuscript to PLOS Computational Biology. After careful consideration, we feel that it has merit but does not fully meet PLOS Computational Biology's publication criteria as it currently stands. Therefore, we invite you to submit a revised version of the manuscript that addresses the points raised during the review process.

Please submit your revised manuscript within 60 days Oct 21 2025 11:59PM. If you will need more time than this to complete your revisions, please reply to this message or contact the journal office at ploscompbiol@plos.org. When you're ready to submit your revision, log on to https://www.editorialmanager.com/pcompbiol/ and select the 'Submissions Needing Revision' folder to locate your manuscript file.

Please include the following items when submitting your revised manuscript:

* A rebuttal letter that responds to each point raised by the editor and reviewer(s). You should upload this letter as a separate file labeled 'Response to Reviewers'. This file does not need to include responses to formatting updates and technical items listed in the 'Journal Requirements' section below.

* A marked-up copy of your manuscript that highlights changes made to the original version. You should upload this as a separate file labeled 'Revised Manuscript with Track Changes'.

* An unmarked version of your revised paper without tracked changes. You should upload this as a separate file labeled 'Manuscript'.

If you would like to make changes to your financial disclosure, competing interests statement, or data availability statement, please make these updates within the submission form at the time of resubmission. Guidelines for resubmitting your figure files are available below the reviewer comments at the end of this letter

We look forward to receiving your revised manuscript.

Kind regards,

Feilim Mac Gabhann, Ph.D.

Editor-in-Chief

PLOS Computational Biology

**Additional Editor Comments (if provided):**

**Journal Requirements:**

If the reviewer comments include a recommendation to cite specific previously published works, please review and evaluate these publications to determine whether they are relevant and should be cited. There is no requirement to cite these works unless the editor has indicated otherwise.

[Note: HTML markup is below. Please do not edit.]

**Reviewers' comments:**

Reviewer's Responses to Questions

**Comments to the Authors:**

**Please note here if the review is uploaded as an attachment.**

Reviewer #1: Solid stresses produced by cancer cell growth in a confined space can compress blood and lymphatic vessels, but the details of the mechanical interactions between cells and structures are poorly understood. Here, the authors present a multiscale agent-based model (ABM) that simulates the mechanical interactions between proliferating tumor cells and vasculature. The authors show how tissue mechanics affect oxygen delivery and vessel remodeling, and conclude that vascular remodeling determines treatment response and post-radiotherapy regrowth.

In general, the study supports well-established findings or previous modeling work regarding the effects of solid stresses. The main innovation is the inclusion of realistic mechanical considerations in an AB model. The assumptions used in the development of the model appear to be sound in terms of the treatment of cell metabolism, cell-cell mechanics and vascular remodeling. I found the model a refreshing departure from the usual continuous models, and believe that such models are more useful for studying tumor heterogeneity. I have a few suggestions for improving the presentation of the study:

Comments:

1) Especially in the introduction, it would be important to make it clear that "pressure" refers to solid pressure, or solid stress, and that fluid pressure is not considered in the model.

2) The authors should clarify what happens at the tumor boundary. Do the cells there experience less friction, or is the constraint imposed by surrounding tissue being represented somehow?

3) An outstanding question in the field relates to how solid stresses are affected by cell invasion (see, e.g. https://doi.org/10.1038/s41551-018-0334-7). Can the model be modified to include active cell migration to address this question?

4) The inclusion of both the drag force and the friction force is confusing. Once the cell is moving, how do these differ, and are they both necessary? Also, Figure 1b could be more detailed to clarify how the friction force is working in the different regimes (stuck vs. moving).

5) Such a model would be straightforward to extend to 3D. The authors should comment on the potential limitations of the 2D simulations.

Reviewer #2: This manuscript presents a novel agent-based computational model of interactions between proliferating tumor cells and the tumor vasculature, focusing on the mechanical forces that are exerted to drive vessel occlusion and pruning due to compression. It also accounts for new vessel growth in response to hypoxia. The model makes novel and non-intuitive predictions about friction forces that represent adhesion between cells and the extracellular matrix. They also predict how dynamics of tumor growth and microvessel remodeling influence response to radiotherapy.

Overall, this is a well written paper that explores interesting biophysical/biomechanical cell-cell and cell-matrix interactions that haven’t yet been evaluated with multi-cell agent-based models. The purpose of the model is well justified, and the model methodology is described in sufficient detail. The quantification of complex spatial structures generated by the agent-based model through the use of spatial statistics is particularly innovative and reveal interesting and important details about tumor and tumor vasculature adaptations over time. I think the paper would be substantially strengthened by making at least a minor attempt at independent model validation by comparison of model predictions to published experimental data. Also, I have some minor comments and questions for the author’s consideration:

1. Methods: Regarding the model’s approximation of “Dynamic Oxygen Supply”, while varying vessel diameters are accounted for, how are the authors accounting for the fact that arterioles, capillaries, and venules contain blood whose oxygen tensions (i.e. P02) differ?

2. Methods: For the “Occlusion, Pruning and Angiogenesis”, the authors are not accounting for the connectivity of the vasculature, such that if one vessels is occluded or pruned, it is likely that nearby “collateral” blood vessels (that are connected to the same upstream parent vessel as the occluded vessel) would experience increased blood flow, and they could dynamically alter their diameter, as a result. In the Discussion, the authors briefly mention this model limitation as a potential opportunity for future model extension, but it would be helpful to also state this important model assumption in the description of the model in the Methods section when it is first described.

3. Methods: It is not clear why each simulation starts with 600 pruned vessels. One would assume that if there are only 4 tumor cells present initially, most if not all of the vessels would be “healthy”.

4. Results: in 3.3 the authors explore how ωh, the threshold oxygen concentration below which cells halt proliferation and become quiescent), affects tumor size, composition and morphology. With lower ωh values, cells can withstand lower oxygen concentrations and remain viable at greater distances from blood vessels, leading to a compact tumor mass. Whereas with higher ωh values, tumor cells are restricted to grow around the perfused blood vessels. They quantify the resulting tumor shape using a roundness score. It would seem that this prediction might be one that has (or could be) quantified in experimental or patient-derived pathology. Validation of this prediction by comparison to existing, publicly available data would strengthen the impact of this discovery.

**********

**Have the authors made all data and (if applicable) computational code underlying the findings in their manuscript fully available?**

The PLOS Data policy requires authors to make all data and code underlying the findings described in their manuscript fully available without restriction, with rare exception (please refer to the Data Availability Statement in the manuscript PDF file). The data and code should be provided as part of the manuscript or its supporting information, or deposited to a public repository. For example, in addition to summary statistics, the data points behind means, medians and variance measures should be available. If there are restrictions on publicly sharing data or code —e.g. participant privacy or use of data from a third party—those must be specified.

Reviewer #1: Yes

Reviewer #2: None

**********

PLOS authors have the option to publish the peer review history of their article (what does this mean?). If published, this will include your full peer review and any attached files.

If you choose “no”, your identity will remain anonymous but your review may still be made public.

**Do you want your identity to be public for this peer review?**  For information about this choice, including consent withdrawal, please see our Privacy Policy.

Reviewer #1: No

Reviewer #2: No

[NOTE: If reviewer comments were submitted as an attachment file, they will be attached to this email and accessible via the submission site. Please log into your account, locate the manuscript record, and check for the action link "View Attachments". If this link does not appear, there are no attachment files.]

**Figure resubmission:**

While revising your submission, we strongly recommend that you use PLOS’s NAAS tool (https://ngplosjournals.pagemajik.ai/artanalysis) to test your figure files. NAAS can convert your figure files to the TIFF file type and meet basic requirements (such as print size, resolution), or provide you with a report on issues that do not meet our requirements and that NAAS cannot fix.

After uploading your figures to PLOS’s NAAS tool - https://ngplosjournals.pagemajik.ai/artanalysis, NAAS will process the files provided and display the results in the "Uploaded Files" section of the page as the processing is complete. If the uploaded figures meet our requirements (or NAAS is able to fix the files to meet our requirements), the figure will be marked as "fixed" above. If NAAS is unable to fix the files, a red "failed" label will appear above. When NAAS has confirmed that the figure files meet our requirements, please download the file via the download option, and include these NAAS processed figure files when submitting your revised manuscript.

**Reproducibility:**

To enhance the reproducibility of your results, we recommend that authors of applicable studies deposit laboratory protocols in protocols.io, where a protocol can be assigned its own identifier (DOI) such that it can be cited independently in the future. Additionally, PLOS ONE offers an option to publish peer-reviewed clinical study protocols. Read more information on sharing protocols at https://plos.org/protocols?utm_medium=editorial-email&utm_source=authorletters&utm_campaign=protocols

# Author comment: Author response to Decision Letter 1

**DOI:** 10.1371/journal.pcbi.1012967.r002

30 Nov 2025

Attachment Submitted filename: Response to Reviewers.pdf

# Peer review: Decision Letter 1

**DOI:** 10.1371/journal.pcbi.1012967.r003

**Authors:** Feilim Mac Gabhann

13 Apr 2026

Dear Mr Fan,

We are pleased to inform you that your manuscript 'Exploring the relationship between vascular remodelling and tumour growth using agent-based modelling' has been provisionally accepted for publication in PLOS Computational Biology.

Before your manuscript can be formally accepted you will need to complete some formatting changes, which you will receive in a follow up email. A member of our team will be in touch with a set of requests.

Please note that your manuscript will not be scheduled for publication until you have made the required changes, so a swift response is appreciated.

IMPORTANT: The editorial review process is now complete. PLOS will only permit corrections to spelling, formatting or significant scientific errors from this point onwards. Requests for major changes, or any which affect the scientific understanding of your work, will cause delays to the publication date of your manuscript.

Should you, your institution's press office or the journal office choose to press release your paper, you will automatically be opted out of early publication. We ask that you notify us now if you or your institution is planning to press release the article. All press must be co-ordinated with PLOS.

Thank you again for supporting Open Access publishing; we are looking forward to publishing your work in PLOS Computational Biology.

Best regards,

Feilim Mac Gabhann, Ph.D.

Editor-in-Chief

PLOS Computational Biology

Feilim Mac Gabhann

Editor-in-Chief

PLOS Computational Biology

***********************************************************

Reviewer's Responses to Questions

**Comments to the Authors:**

**Please note here if the review is uploaded as an attachment.**

Reviewer #1: The authors have sufficiently addressed my concerns.

**********

**Have the authors made all data and (if applicable) computational code underlying the findings in their manuscript fully available?**

The PLOS Data policy requires authors to make all data and code underlying the findings described in their manuscript fully available without restriction, with rare exception (please refer to the Data Availability Statement in the manuscript PDF file). The data and code should be provided as part of the manuscript or its supporting information, or deposited to a public repository. For example, in addition to summary statistics, the data points behind means, medians and variance measures should be available. If there are restrictions on publicly sharing data or code —e.g. participant privacy or use of data from a third party—those must be specified.

Reviewer #1: Yes

**********

PLOS authors have the option to publish the peer review history of their article (what does this mean?). If published, this will include your full peer review and any attached files.

If you choose “no”, your identity will remain anonymous but your review may still be made public.

**Do you want your identity to be public for this peer review?**  For information about this choice, including consent withdrawal, please see our Privacy Policy.

Reviewer #1: No

# Editor report: Acceptance letter

**DOI:** 10.1371/journal.pcbi.1012967.r004

**Authors:** Feilim Mac Gabhann

PCOMPBIOL-D-25-00500R1

Exploring the relationship between vascular remodelling and tumour growth using agent-based modelling

Dear Dr Fan,

I am pleased to inform you that your manuscript has been formally accepted for publication in PLOS Computational Biology. Your manuscript is now with our production department and you will be notified of the publication date in due course.

The corresponding author will soon be receiving a typeset proof for review, to ensure errors have not been introduced during production. Please review the PDF proof of your manuscript carefully, as this is the last chance to correct any errors. Please note that major changes, or those which affect the scientific understanding of the work, will likely cause delays to the publication date of your manuscript.

Soon after your final files are uploaded, unless you have opted out, the early version of your manuscript will be published online. The date of the early version will be your article's publication date. The final article will be published to the same URL, and all versions of the paper will be accessible to readers.

For Research, Software, and Methods articles, you will receive an invoice from PLOS for your publication fee after your manuscript has reached the completed accept phase. If you receive an email requesting payment before acceptance or for any other service, this may be a phishing scheme. Learn how to identify phishing emails and protect your accounts at https://explore.plos.org/phishing.

Thank you again for supporting PLOS Computational Biology and open-access publishing. We are looking forward to publishing your work!

With kind regards,

Judit Kozma

PLOS Computational Biology | Carlyle House, Carlyle Road, Cambridge CB4 3DN | United Kingdom ploscompbiol@plos.org | Phone +44 (0) 1223-442824 | ploscompbiol.org | @PLOSCompBiol
